# Analytical pipeline optimisation in developmental fNIRS hyperscanning data: Neural coherence between 4- to 6-year old children collaborating with their mothers

**DOI:** 10.1162/imag_a_00509

**Published:** 2025-03-20

**Authors:** Victoria St. Clair, Letizia Contini, Rebecca Re, Paola Pinti, Denis Mareschal

**Affiliations:** Centre for Brain and Cognitive Development, Birkbeck, University of London, London, United Kingdom; Politecnico di Milano, Dipartimento di Fisica, Piazza Leonardo da Vinci, Milan, Italy; Istituto di Fotonica e Nanotecnologie, Consiglio Nazionale delle Ricerche, Piazza Leonardo da Vinci, Milan, Italy; Department of Medical Physics and Biomedical Engineering, University College London, London, United Kingdom

**Keywords:** functional near-infrared spectroscopy, hyperscanning, neural synchrony, wavelet transform coherence, collaborative problem solving, parent–child interaction

## Abstract

Much of a child’s early learning takes place during social interactions with others. Neural synchrony, the temporal alignment of individuals’ functional brain activity, is a neural mechanism that may support successful interaction, but its biological origins and sensitivity to environmental factors remain unknown. This study measures neural coherence between 4- to 6-year-old children and their mothers using wearable functional near-infrared spectroscopy (“fNIRS”) in a collaborative problem-solving hyperscanning paradigm. Best practices in fNIRS data processing are incorporated to optimise coherence quantification and extricate environmental- and task-related effects. Results suggest physiological noise in the extracerebral layer artificially inflated coherence strength in both oxygenated (“HbO_2_”) and deoxygenated (“HbR”) haemoglobin. Coherence remained stronger during collaborative than during individual problem solving in both chromophores after physiological noise reduction. Phase-scrambled pseudodyad analyses supported the interpretation that coherence during collaboration relates to temporal dynamics of interaction rather than to task- or environmental-related components. Strength of HbO_2_coherence was positively related to collaborative task performance and negatively related to background maternal stress. HbR coherence was also related to task performance and maternal stress but the direction of results were mixed. Overall, this study provides new insight into the nature of neural coherence between 4- to 6-year-old children and their mothers during collaborative play.

## 1 Introduction

Humans are a fundamentally social species. In young life, much of a child’s learning occurs during interaction with parents, siblings, and peers. One possible neural process underlying successful interaction is that of neural synchrony, defined broadly as the temporal alignment of neural activity between two or more people (Wass et al., 2020). A small number of developmental studies have found that synchrony is elicited during children’s interactions with others; however, there is a need to replicate initial results using rigorous analytical pipelines to process dual-brain neuroimaging data in children. This study applies best practices in treatment of functional near-infrared spectroscopy (“fNIRS”) hyperscanning data to test the emergence of neural coherence between 4- to 6-year-old children and their mothers during children’s collaborative interactions.

### 1.1 Hyperscanning with fNIRS

fNIRS is a non-invasive neuroimaging technology that uses near-infrared light to measure changes in oxygenated (“HbO_2_”) and deoxygenated (“HbR”) haemoglobin over the cortical surface (seePinti et al., 2020,^2023^). fNIRS allows for inference of neural activity from indirect measurements of changes in HbO_2_and HbR known to follow neuronal firing (neurovascular coupling;Moradi & Buxton, 2013). fNIRS has been used successfully with children and in challenging and unconventional experimental contexts, such as in rural settings (Lloyd-Fox et al., 2014), during sleep (Taga et al., 2018), and in virtual reality environments (Bulgarelli et al., 2023). fNIRS is also used to measure brain activity from more than one individual simultaneously in hyperscanning designs. Unlike single-brain studies, dual-brain recordings can quantify the continuous coordination of two individuals’ functional brain activity that may arise from mutual interaction (Konvalinka & Roepstorff, 2012). Overall, fNIRS is well suited for investigating neural activity during naturalistic social interactions (Greaves et al., 2022;Minagawa et al., 2018;Pinti et al., 2018).

The most common method for calculating the alignment of brain activity from hyperscanning data is wavelet transform coherence (“WTC”; e.g.,Cui et al., 2012;Dommer et al., 2012;Hakim et al., 2023;Hirsch et al., 2017,^2018^;X. Zhang et al., 2020). The WTC calculation decomposes neural signals from the time domain into the time-frequency domain with the wavelet transform (Torrence & Compo, 1998) and calculates coherence between the two signals, independent of constant phase shift, at each frequency over time. Coherence can then be averaged across frequency bands that contain the haemodynamic response of interest (Hakim et al., 2023) and over experimental time (e.g., within condition). The result is a measure that represents the temporal and spectral relationship between two individuals’ neural signals or, more specifically in the case of fNIRS, between two participants’ relative changes in concentrations of HbO_2_and HbR in cortical tissue.

### 1.2 Synchrony during collaborative problem solving

Neural synchrony emerges during social interaction (Babiloni & Astolfi, 2014;Czeszumski et al., 2022;Nguyen et al., 2024); however, there are many, non-mutually exclusive explanations of its causes (e.g., seeHamilton, 2021;Hoehl et al., 2021;Holroyd, 2022;Kingsbury et al., 2019). Broadly, the brain utilises patterns in stimuli, such as frequency and temporal rhythm (e.g., of speech,A. Giraud & Poeppel, 2012) to encode information from the environment (stimulus-to-brain coupling;Buzsaki, 2006). In social environments, how stimuli is encoded is affected by the presence of others, which may give rise to brain-to-brain coupling between individuals (Dumas, 2011;Hasson et al., 2012). Interacting with someone requires mentally representing the other person and inferring their shifting internal states, such as their goals and beliefs, in relation to their behaviours in real time as an interaction unfolds (C. D. Frith & Frith, 1999;U. Frith & Frith, 2001). Both partners must also contingently respond to the other person in a reciprocal process of adaptation, interpreting and predicting the actions of the other while planning and executing their next actions. The neural substrates of these social cognitive processes are thought to originate in the prefrontal and temporoparietal cortices, and functional brain activity in these regions is thought to align between interacting individuals during collaborative tasks, at least in adults (Czeszumski et al., 2022;Hoehl et al., 2021;Lotter et al., 2023;Wang et al., 2022).

Several studies have investigated neural coherence in children and their parents during collaborative tasks (Kruppa et al., 2020;Miller et al., 2019;Nguyen et al., 2020;Nguyen, Schleihauf, et al., 2021;Reindl et al., 2018).Reindl et al. (2018)found evidence of coherence in HbO_2_during cooperative but not competitive computer game play between dyads of 5- to 9-year-old children playing with their mothers or fathers. The effect was driven by higher synchrony in the left prefrontal cortices during cooperation compared with competition.Miller et al. (2019)showed that coherence between 8- to 12-year-old children and their parents in HbO_2_was significantly higher in cooperation compared with independent conditions with a computer-based task similar toReindl et al. (2018). Analysis was conducted across five regions of interest (“ROI”), such that WTC was calculated for each matched channel pair (e.g., child channel 10 to parent channel 10) and pairs’ WTC values were averaged within ROI. Results did not reveal a main effect of ROI and, while uncorrected post hoc comparisons suggested coherence was higher in the right dorsolateral and frontopolar regions in cooperation than in independent conditions, no significant comparisons within regions survived FDR correction.Kruppa et al. (2020)also reported increased neural synchrony, particularly in the frontopolar cortex, during cooperation compared with competition among 8 to 18 year olds.

Building on this work, later research implemented cooperative tasks with younger, 5- to 6-year-old children and their parents (Nguyen et al., 2020;Nguyen, Schleihauf, et al., 2021) using a physical Tangram puzzle-solving task that allowed children and parents to play together naturally. The results showed that coherence during cooperative problem solving was higher than during individual problem solving and rest (Nguyen et al., 2020;Nguyen, Schleihauf, et al., 2021), a finding also replicated byNguyen et al. (2024). In summary, there is a limited yet promising evidence base suggesting that neural coherence emerges more strongly during collaborative than during independent tasks between children and their parents; however, important, unanswered questions remain about the role of non-cerebral signal alignment and task-related activation in the calculation of coherence.

### 1.3 Investigating origins of synchrony

When inferring brain activity or brain-to-brain coupling from fNIRS signals, confounding factors that can compromise the robustness of fNIRS data must be taken into account to avoid false positives and false negatives (Tachtsidis & Scholkmann, 2016;Yücel et al., 2021). One intrinsic limitation of fNIRS is that the NIR light must pass through different layers of tissue (e.g., through the scalp) before reaching the cortex. Fluctuations in haemoglobin concentrations in non-cortical tissue are known to be sensitive to participants’ systemic physiology, such as changes in heart rate, breathing rate, or blood pressure (Franceschini et al., 2006). These can contaminate fNIRS signals, which are generally interpreted as representing cortical haemodynamic activity. Research also shows that physiological rhythms, such as heart rate (McCraty, 2017) and breathing rhythms (Konvalinka et al., 2022), synchronise between individuals during interaction. Whether non-neural signals and/or their alignment affects strength of neural coherence between children and their parents during collaborative tasks has yet to be explored.

Another challenge is that most fNIRS hyperscanning research on collaborative problem solving in children only reports coherence in changes in concentrations of HbO_2_, which is known to be more sensitive to non-cortical changes in participant physiology than HbR.Tachtsidis and Scholkmann (2016)argue that reporting concentration changes only in HbO_2_may lead to false positives, where changes in haemodynamic activity are attributed to functional brain activity when they are instead related to non-neuronal, systematic changes in physiology (e.g., changes in respiration or in blood pressure). In a systematic review of 660 fNIRS studies,Kinder et al. (2022)found that approximately half report results in HbO_2_only, without providing a justification. Similarly toTachtsidis and Scholkmann (2016),Kinder et al. (2022)suggest that reporting data from only one chromophore can lead to misinterpretations of results because the inverse correlation of chromophores cannot always be assumed. Reporting both chromophores can facilitate detection of unexpected influences on fNIRS signal (e.g., non-neuronal artefacts;Kinder et al., 2022). This study (i) implements superficial signal regression using short separation channels to assess the impact of physiological noise reduction from the cortical signal of interest on coherence during collaborative problem solving and (ii) reports results in both oxygenated and deoxygenated haemoglobin.

Theoretical explanations about why synchrony emerges during social interaction also require further development. For example, coordination of eye-gaze patterns and direct eye contact with a communicative partner is thought both to facilitate smooth interaction and support the ability to infer or predict a person’s mental states (Baron-Cohen, 2010). Some suggest synchrony is related to attachment quality (Nguyen et al., 2024), children’s irritability (Quiñones-Camacho et al., 2020), positive value attribution (Hoehl et al., 2021), or proximity to and touch from caregivers (Nguyen, Abney, et al., 2021). It also appears to be the case that eye contact relates to strength of inter-brain synchrony during interaction (Dravida et al., 2020;Hirsch et al., 2017;Noah et al., 2020;Saito et al., 2010, though seeHaresign et al., 2023). Using fNIRS with adults,Hirsch et al. (2017)reported coherence in HbR across prefrontal and temporoparietal regions was stronger during an eye-to-eye contact than during an eye-to-picture condition. Extending these findings,Noah et al. (2020)found that synchrony in HbO_2_and HbR between interacting adults’ temporoparietal junctions was also higher during in-person eye-to-eye contact than during viewing of dynamic videos of faces making direct eye contact.

Evidence from EEG with 9-month-old infants interacting with their parents suggests direct eye contact increases inter-brain coupling (Leong et al., 2017), thoughHaresign et al. (2023)did not replicate this result. Further, behavioural research on gaze coordination during free play has suggested that, especially as children get older, manipulation of objects in the shared environment might underlie states of joint attention between parents and children (Franchak et al., 2024;Sun et al., 2024;Yu & Smith, 2017). The current study tests whether the strength of neural synchrony in visually unobstructed collaboration is higher than in a partial collaboration condition, where an opaque half-curtain is placed between participants and prevents their ability to make direct eye contact while working together.

It is also possible that synchrony arises simply because two individuals are in the same physical environment, looking at common stimuli and doing similar tasks, which causes common bottom-up perceptual processing. Previous research has attempted to rule out this explanation using different experimental manipulations, such as including individual task conditions in which participants perform the same task as is done collaboratively but without the social interaction (e.g.,Nguyen et al., 2020) or by introducing both participants to the same visual stimuli, such as watching a video together without interacting (e.g.,Nguyen, Abney, et al., 2021). Completing a task on one’s own removes social context, including ostensive behavioural cues (e.g., eye gaze,Hirsch et al., 2017) and prevents the possibility of mutual prediction (Kingsbury et al., 2019;Mayo & Shamay-Tsoory, 2024). Analytic techniques can also be used to rule out spurious and/or task-related elements of neural synchrony. For example, data from “pseudodyads” can be generated by either partner scrambling (e.g.,Hirsch et al., 2017;Nguyen et al., 2020;Reindl et al., 2018) or phase scrambling (e.g.,Piazza et al., 2020;Yang et al., 2021). Partner scrambling analyses synchrony between two individuals who completed the same task but who did not complete it together (e.g., child A to mother B, child B to mother C, etc.). If synchrony reflects similarity in cognitive operations, regardless of interactive partner, it should be equally strong in both true dyads and pseudodyads who are all performing a similar task. However, if synchrony reflects a process specific to one’s direct interaction with another person, then synchrony ought to be significantly stronger in the true dyads than in the pseudodyads. For example,Hirsch et al. (2017)found significant cross-brain coherence in true dyads but not in partner-scrambled dyads, which supports an interpretation of cross-brain coherence as related to specific interactive events rather than common cognitive processing. Partner scrambling requires the timeline of the conditions to be the same across dyads so that data from child A temporally match with the data from mother B. However, this is not always possible nor desirable for unstructured and naturalistic experiments, where the onsets of the conditions can vary across dyads. One benefit of naturalistic paradigms is that they preserve as much ecological validity as possible and are not as tightly controlled as traditional computer-based tasks. Phase scrambling may be a more suitable option than partner scrambling for naturalistic hyperscanning data as it allows for the preservation of temporal alignment of two participants within a dyad across a sample with heterogeneous experimental timelines.

Phase scrambling uses a fast Fourier transform to extract the magnitude and phase of the data from one of the two interacting partners. Then, an inverse fast Fourier transform is used to reconstruct the surrogate data using the original magnitude and a randomised phase (Simony et al., 2016). This procedure preserves the frequency (e.g., mean and autocorrelation) of the original signal but disrupts any meaningful temporal information (Piazza et al., 2020). Coherence values can be calculated between the child’s original data and several iterations of the mother’s phase-scrambled data capture synchrony between the two individuals in terms of signal frequency but not time frequency. Phase-scrambled WTC data can, therefore, be interpreted as neural synchronisation expected for frequencies measured in the same experimental conditions, but without considering the temporal nature of direct interpersonal interaction. By comparing true dyad with pseudodyad coherence values, it is possible to test whether synchrony is significantly higher in signals that account for the temporal nature of online interaction over-and-above task- or environmental-related frequency alignment. Here, a phase-scrambled approach is used to investigate whether coherence measurement arises from a shared physical environment and task or from coordinated social interaction. If synchrony is a phenomenon specific to social engagement, it is expected to be stronger in true dyads than in phase-scrambled dyads during the collaboration conditions.

### 1.4 Synchrony and task performance

Evidence about whether neural synchrony predicts behavioural cooperative problem-solving performance is mixed (adults:Baker et al., 2016;Cui et al., 2012; children:Miller et al., 2019;Nguyen et al., 2020;Nguyen, Schleihauf, et al., 2021;Reindl et al., 2018).Reindl et al. (2018)found relationships between coherence and performance in some analyses but not in others, whileNguyen et al. (2020)report a positive relationship between coherence in collaborative problem solving and task performance between mothers and children.Nguyen, Schleihauf, et al. (2021)do not replicate this relationship between fathers and children. Other research has failed to replicate the expected relationship (Miller et al., 2019;Reindl et al., 2022). In 8- to 12-year-old children,Miller et al. (2019)did not report robust relationships between task performance and coherence during cooperative game play.Reindl et al. (2022)also did not find significant relationships between neural synchrony and cooperative task performance in 10- to 18-year olds and mothers. There is a clear need to replicate previously reported effects to investigate whether the relationships between synchrony and behavioural performance on collaborative tasks are robust.

### 1.5 Synchrony and maternal stress

Some research has found that maternal background factors, such as stress and anxiety levels, are related to synchrony between parents and children during interaction (Azhari et al., 2019;Nguyen et al., 2020;Papoutselou et al., 2022;Smith et al., 2022, but seeNguyen, Schleihauf, et al., 2021). One study tested whether neural synchrony between 4- and 5-year-old children and their primary caregivers during problem solving was sensitive to experimentally induced environmental stress (Hoyniak et al., 2021). Dyads completed Tangram puzzles in conditions that were either stressful (i.e., timed or containing difficult puzzles and/or distracting toys present) or non-stressful (e.g., untimed or containing easier puzzles and/or no distracting toys present). During the stressful conditions, dyads showed reduced neural synchrony over the lateral prefrontal cortex (“PFC”) compared with non-stressful conditions (Hoyniak et al., 2021). Using a parent-report background measure of maternal stress,Nguyen et al. (2020)found that mothers with higher stress levels showed weaker neural synchrony with their children over the temporoparietal and prefrontal areas during collaborative problem solving than did mothers with lower general self-report stress levels (Nguyen et al., 2020; though a similar effect was not reported in child–father dyads,Nguyen, Schleihauf, et al., 2021). It may be the case that both situational and background stress have a dampening effect on neural synchrony during 4- to 6-year-old children’s collaborative problem solving.

### 1.6 Present study

This study applies best practices to the analysis of developmental fNIRS hyperscanning data to assess the resilience of previously reported effects in coherence during children’s interactions. Specifically, this study aims to (i) use physiological noise reduction techniques to assess the extent to which coherence in HbO_2_and HbR may be artificially inflated by alignment of activity in the extracerebral layer; (ii) test whether, after reduction of physiological noise, neural coherence between children and their mothers emerges more strongly during collaborative than during individual problem solving; (iii) assess whether the availability of the partner’s face information relates to the strength of coherence; (iv) rule out the possibility that coherence is entirely explained by alignment in task- or environmental-related frequency components; and (v) examine whether strength of coherence relates to variables in the behavioural domain.

## 2 Method

### 2.1 Participants

Data from*N*= 47 dyads of mothers and their 4- to 6-year-old children (21 females, age mean ± SD = 5.11 ± 0.83 years, range = 4.01–6.90 years) were collected at Birkbeck, University of London’s ToddlerLab. Data from a further*n*= 13 participants were collected, but*n*= 1 attended with father,*n*= 5 refused the fNIRS cap,*n*= 5 were excluded for technical and/or experimenter error, and*n*= 2 were excluded for failure to meet data quality inclusion criteria for channel- and ROI-wise analysis (defined as at least one valid region of interest per participant, with valid regions defined as a region with at least two valid long separation channels, “LSCs”). Participants were recruited from a pre-existing database of volunteers at Birkbeck’s Centre for Brain and Cognitive Development. All children were typically developing and born at term. Mothers provided written informed consent for themselves and their children before data were collected in pre-laboratory questionnaires and in-laboratory experimental protocol. This study was approved by the Ethics Committee of the Department of Psychological Sciences at Birkbeck, University of London (approval number: 2122046).

### 2.2 Procedure

#### 2.2.1 Parental stress scale

Before the laboratory visit, mothers received a RedCap link containing a consent form and a Parental Stress Scale (“PSS”,Berry & Jones, 1995) to complete 1 week before their laboratory visit. The PSS is an 18-item self-report questionnaire that asks parents to rate their agreement with statements about the positive and negative experiences of parenting on a 5-point Likert scale ranging from “Strongly disagree” to “Strongly agree.”

#### 2.2.2 Experimental protocol

**Fig. 1.**
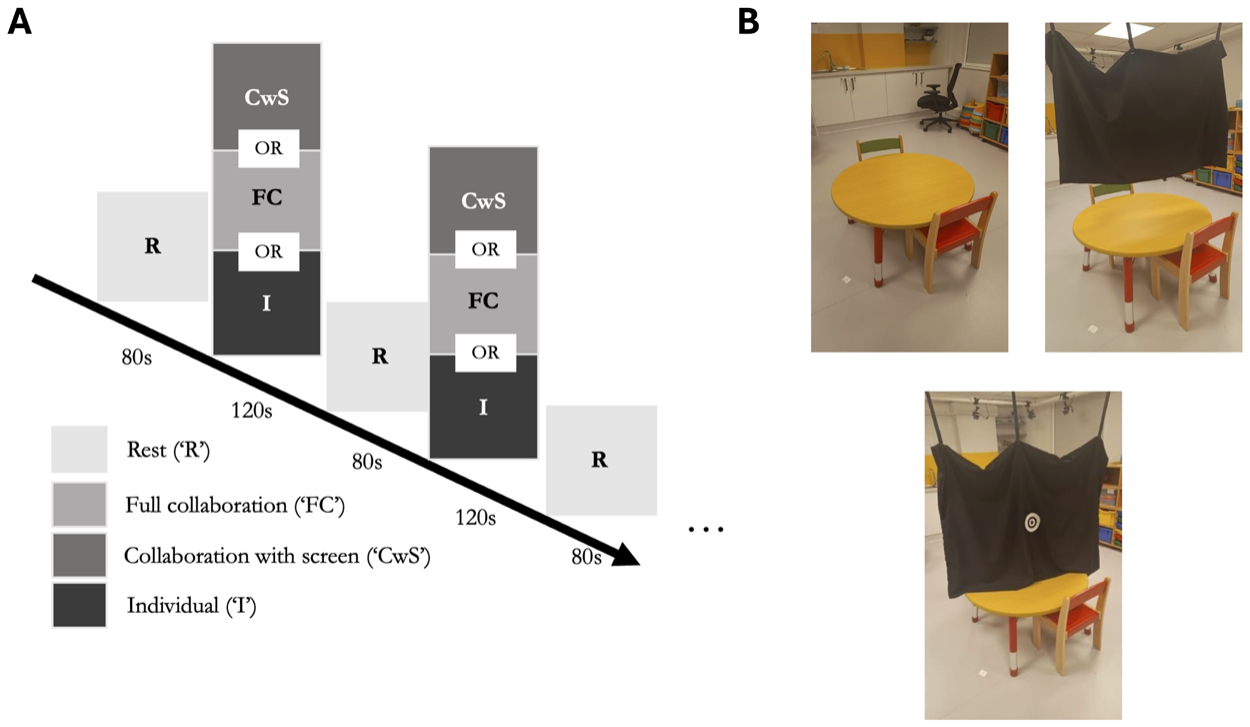
Experimental protocol and conditions. (A) Schematic of protocol. All sessions began and ended with a rest phase (80 seconds), and the order of experimental conditions—full collaboration, collaboration with screen, and individual—was randomised. There were three trials per condition. (B) Depiction of full collaboration, collaboration with screen, and individual conditions.

Mothers and children were invited to the Birkbeck ToddlerLab in Central London. After written informed consent was obtained for both mothers and children, the dyad was asked to sit face-to-face on either side of a child-sized table (seeFig. 1). Dyads arranged Tangram puzzles according to different templates (abstract forms, objects, animals, etc) replicating the task implemented byNguyen et al. (2020). Each task phase contained 120-second task phases of 3 conditions: individual, collaboration with screen, and full collaboration (Fig. 1).

In the full collaboration condition, dyads were asked to work together to complete the Tangram puzzles as specified by the templates provided. In the collaboration with screen condition, a short curtain was drawn so they could work together while viewing each other’s hands but not each other’s faces. They were again asked to work together to solve the puzzle. In the individual condition, a full curtain was drawn between participants, and they were asked to solve their puzzles separately and independently. Three trials of each condition were presented in random order and separated by 80-second rest phases. During the rest phase, the long curtain was drawn; participants were asked to close their eyes and rest to prepare for the next puzzle. Some children struggled to stay still and quiet during rests, so were given the opportunity to watch a non-social video of outdoor scene footage (e.g., mountains, seas). A computer script prompted the experimenter to start and end each phase in the order that has been randomly assigned.

### 2.3 Task performance

In each condition, the number of templates parents and children were given by the experimenter, the number they indicated having completed, and the number they completed correctly were tallied. Task performance coding was completed offline using behavioural video recordings.

### 2.4 fNIRS data acquisition

Two continuous-wave, wearable and mobile fNIRS systems—one for the mother and one for the child—were used (Brite MKII, Artinis Medical Systems BV, The Netherlands). Each cap held 16 long separation channels measuring HbO_2_and HbR concentration changes over the left and right prefrontal cortex and left and right temporoparietal junction (seeFig. 2). These areas have been identified in previous research as supporting social mentalising in a cooperative setting (Jiang et al., 2012;Liu et al., 2016;Lotter et al., 2023;Miller et al., 2019;Nguyen et al., 2020;Redcay & Schilbach, 2019;Reindl et al., 2018). Each NIRS system was equipped with eight light emitting diode fibres (sources, 760 and 850 nm) and eight photodiodes (detectors). Raw intensity signals at the two wavelengths were recorded at a sampling frequency of 25 Hz. Source-detector separation was 30 mm for mothers and 25 mm for children. Each system also included two short separation channels (“SSCs”, 10 mm source–detector separation), one located on left PFC and one on right TPJ, to sample blood flow changes in the superficial layers of the scalp in different regions to account for the heterogeneity of extracerebral components (Gagnon et al., 2012;Wyser et al., 2020).

**Fig. 2.**
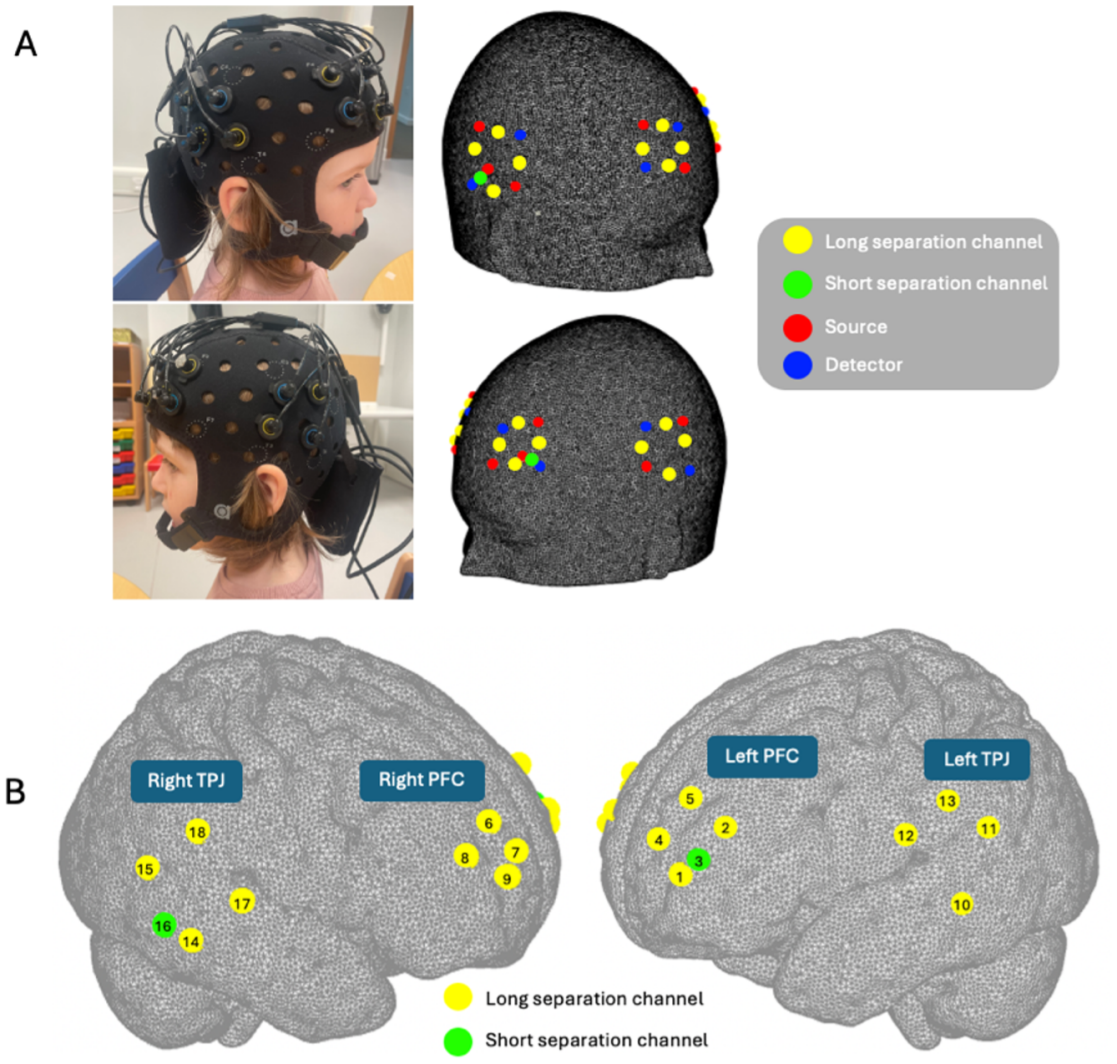
Depiction of optode and channel configuration. (A) Visualisation of cap, optode, and channel configuration. (B) Depiction of specific channel locations in four regions of interest. SeeTable S1for MNI coordinates and LONI Probabilistic Brain Atlas labels. Yellow = long separation channel, green = short separation channel, red = source, and blue = detector.

The 10-20 EEG electrode placement system was used to ensure fNIRS caps were placed reliably across participants. The Fpz point marked on the cap was aligned with the participant’s Fpz point, and the border of the cap was parallel to the eyebrows (for more seePinti et al., 2015). Montreal Neurological Institute coordinates and anatomical locations of each channel are listed inTable S1.

### 2.5 Data pre-processing

#### 2.5.1 Questionnaire data

The sum of parent’s ratings for the items on the Parental Stress Scale (Berry & Jones, 1995) was calculated. Outliers were defined as values that fell 2 standard deviations above or below the mean, and three outliers were removed for analysis.

#### 2.5.2 fNIRS data

fNIRS data were pre-processed using the Homer2 toolbox (Huppert et al., 2009) and following the pre-processing steps for similar study designs (Pinti et al., 2018). The raw intensity signals were visually inspected to identify channels with a low signal-to-noise ratio because of detector saturation, poor optical coupling (e.g., without a clear heart-rate peak around 1.5–2 Hz), or substantial movement artefacts. Compromised channels were excluded from further analyses. SeeTable S1for a full list of the number of participants with valid data per channel. Raw intensity signals were then converted into changes in optical density (Homer2 function,*hmrIntensity2OD*). Motion artefacts were corrected using the wavelet-based method (Molavi & Dumont, 2012; Homer2 function:*hmr MotionCorrect-Wavelet;*interquartile range = 1.5 for mothers and 0.8 for children, seeDi Lorenzo et al., 2019;Pinti et al., 2024). A band-pass filter was applied to remove high-frequency noise such as heart beats and slow drifts (Homer2 function:*hmr BandpassFilt*; order, third; band-pass frequency range [0.01 0.5] Hz;Nguyen et al., 2020). Pre-processed optical density signals were converted into concentration changes in HbO_2_and HbR using the modified Beer–Lambert law (Homer2 function:*hmrOD2Conc*). Differential pathlength factor (“DPF”) values were calculated using system wavelength values of 760 and 850 nm, and the different optical properties of the two age groups were accounted for using an age correction. A DPF of 5.4 and 4.7 was used for children and of 6 and 6 for mothers (Scholkmann & Wolf, 2013).

##### 2.5.2.1 Reduction of physiological noise

Physiological confounds were reduced in the fNIRS signal of interest before analysis. False positives in fNIRS analysis likely arise from the presence of systemic physiological changes, such as changes in heart rate, blood pressure, and breathing, many of which drive haemodynamic changes in the extracerebral layer (Tachtsidis & Scholkmann, 2016). These changes are present in the signal but are not related to neural activity of interest (Tachtsidis & Scholkmann, 2016) and, depending on the cap design, may be 10 to 20 times higher than haemodynamic changes in the cortex (Al-Rawi et al., 2001;Liebert et al., 2004).

Because extracerebral changes have been shown to be spatially heterogeneous across the scalp (Kirilina et al., 2012,^2013^;Näsi et al., 2013;Y. Zhang et al., 2015), SSCs were placed in both the prefrontal cortex and temporoparietal junction regions. A global superficial signal regression (“SSR”) was used to regress average short separation signal out of each signal from long separation channels to reduce physiological noise in the extracerebral layer (Goodwin et al., 2014;Li et al., 2021;Pinti et al., 2024). This allowed for precise analysis of coherence in changes in haemoglobin concentrations in cortical tissue. Previous research using four SSCs shows little to no difference between global versus local regression methods for developmental fNIRS data (Pinti et al., 2024). In the present study, each array contained two SSCs; therefore, global regression was preferable to local regression to use all available SSCs without compromising SSR specificity.

##### 2.5.2.2 Wavelet transform coherence analysis

In line with previous research, neural synchrony was calculated with WTC using the*Cross Wavelet and Wavelet Coherence*toolbox in MATLAB (Grinsted et al., 2004; for details about wavelet transform, seeTorrence & Compo, 1998). Before WTC was calculated, time stamps of fNIRS samples and condition triggers were checked to ensure synchronisation of the two NIRS systems was exact. WTC was calculated for every possible combination of child-to-mother’s long separation channels, excluding short separation channels and noisy channels identified manually. For region of interest analysis, signal from channels within each of the four ROIs was averaged across experimental time before WTC was calculated on every possible combination of child-to-mother’s ROIs.

For both channel-wise and ROI-wise data, coherence was averaged across the period of interest, identified in previous research as 0.02–0.10 Hz (Nguyen et al., 2020;Nguyen, Schleihauf, et al., 2021), and averaged within experimental condition. Dyads who complete at least two experimental trials per condition were included in channel- and ROI-wise analysis. Valid ROIs were defined as regions containing at least two valid long separation channels (Nguyen et al., 2020). Both participants in the dyad needed to contribute at least one valid ROI to be included in both channel- and ROI-wise analyses.

**Fig. 3.**
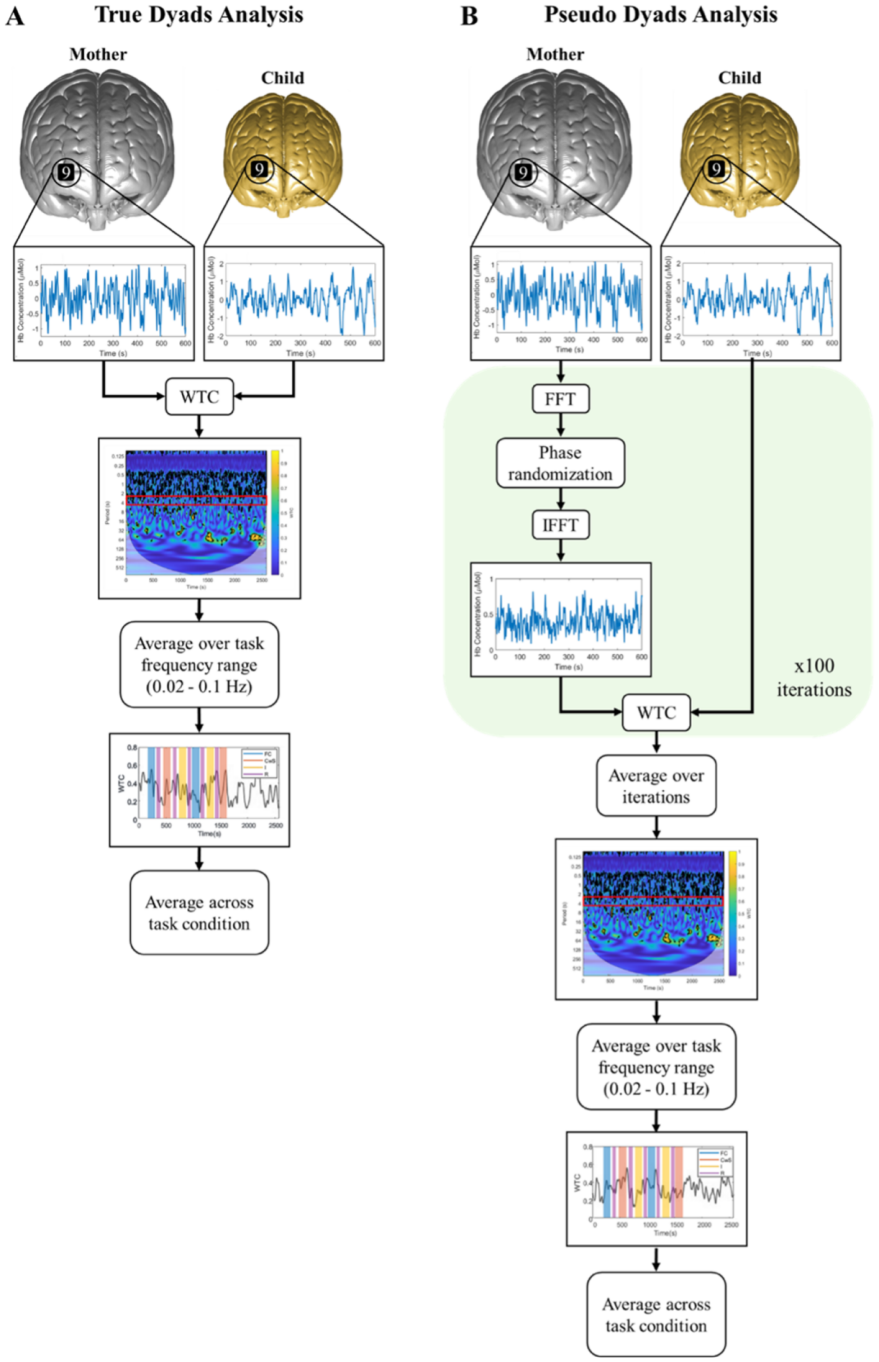
Depiction of WTC analysis pipeline for (A) true dyads and (B) pseudodyads. Fast Fourier transform (“FFT”), inverse fast Fourier transform (“IFFT”), and Wavelet Transform Coherence (“WTC”) were all computed in MATLAB 2022a.

For each dyad, fNIRS signals from the mother were phase scrambled in 100 iterations (Simony et al., 2016). In this process, a fast Fourier transform is used to extract the magnitude and phase of the signal before an inverse fast Fourier transform is used to reconstruct surrogate data with the original magnitude and phase. For each iteration, coherence was calculated between the child’s true signal and the mother’s phase-scrambled signal. Coherence between the true child’s signal and each of the 100 phase-scrambled signals from the mother was then averaged within dyad for every possible combination of both channel-wise and ROI-wise pairing. This resulted in a pseudodyad distribution against which true dyad synchrony values were compared across experimental conditions.Figure 3illustrates the WTC analysis pipeline for one channel-to-channel combination for both true dyads (Fig. 3A) and pseudodyads (Fig. 3B). Data were exported for statistical analysis in R version 4.2.2 (R Core Team, 2021). WTC pipeline and analysis scripts are publicly available athttps://github.com/vmousley/pc_fNIRS_hyperscanning.

## 3 Results

Analysis was conducted separately for both HbO_2_and HbR. For all models, the outcome was neural coherence, and a random effect of participant was included. Model complexity increased in a stepwise fashion and was compared with either a null model—*m*0: WTC ~ (1|id)—or to the best fit model in the sequence using Likelihood Ratio Tests.

### 3.1 Aim 1: reduction of physiological noise

WTC in both chromophores was calculated first in data without SSR, then again after SSR. Coherence was aggregated across all trials at the channel-wise level. Paired, one-sided Welch’s*t*-tests were used to test the effect of SSR on average values of coherence in each condition. Average coherence in HbO_2_was significantly lower after physiological regression within all three experimental conditions. In HbR, synchrony values were significantly lower after SSR in both collaboration conditions but not in the individual condition (*p*= .207, seeFigure 4,Table S2, andTable S3). The direction of primary results (Aim 2) did not differ in data with versus without SSR, but effects were slightly stronger in data where systemic contamination had been reduced (Table S6).

**Fig. 4.**
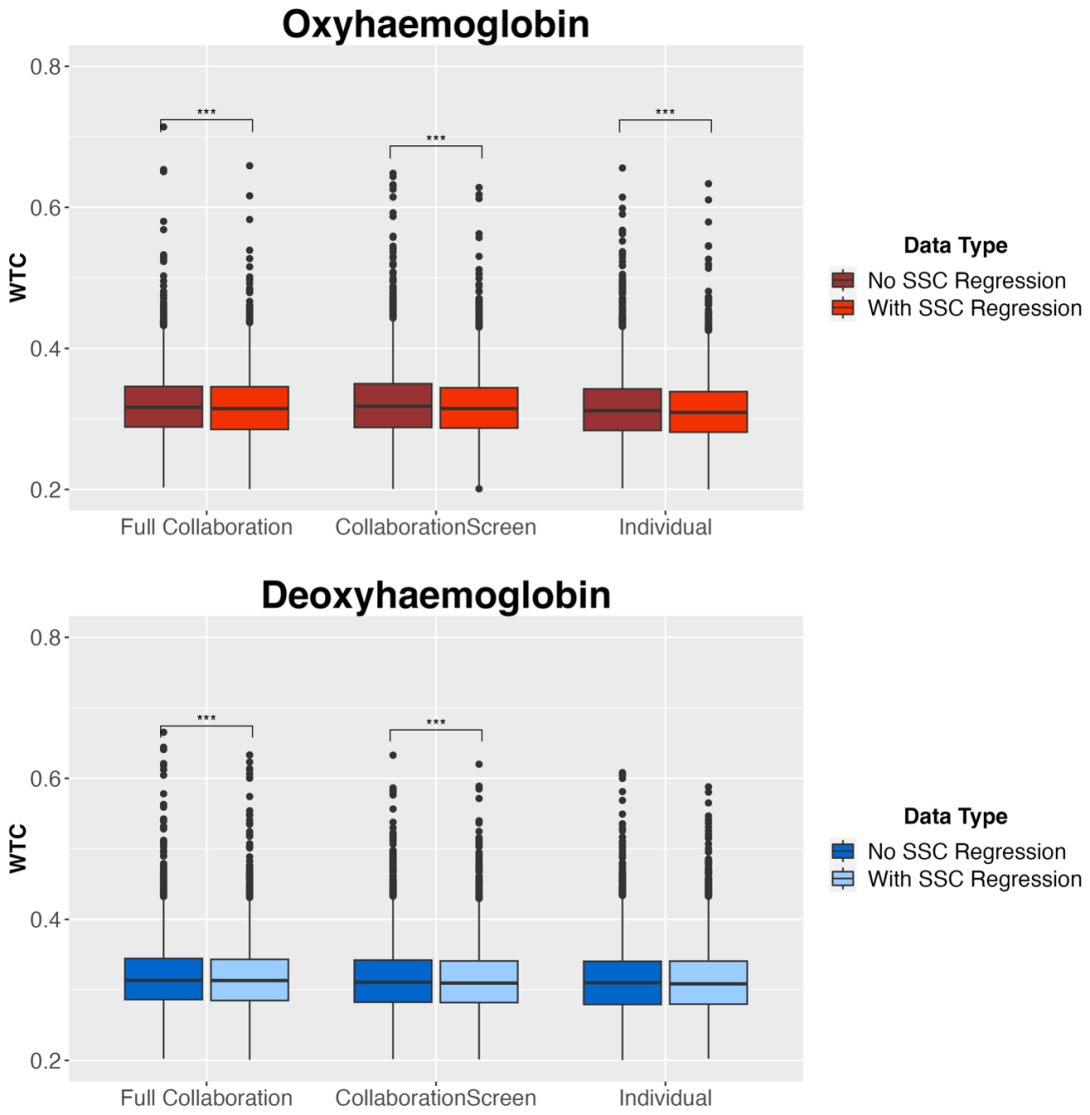
Impact of SSR on synchrony strength by condition. Results of one-way, paired Welch’s*t*-tests for*n*= 38 dyads who contributed data both with and without SSR for HbO_2_and HbR by experimental condition. Coherence values averaged across experimental trials. Bars represent standard error. ***p< .001.*p*-values FDR corrected for multiple comparisons.

### 3.2 Aim 2: coherence in collaboration compared with individual problem solving

To retain as much data as possible, all consequent analyses were conducted on a sample of*n*= 47 datasets in which*n*= 38 had at least one valid short separation channel that was used to regress extracerebral noise out of long separation channels and*n*= 9 contributed unregressed data. Including SSR where possible reduced the impact of scalp blood flow on brain activity estimation in the primary analyses (Goodwin et al., 2014). Effects of age, trial number, and data type (with versus without SSR) were added sequentially to check for confounding effects on neural synchrony in both HbO_2_and HbR. Any term that significantly improved model fit was added as a covariate in subsequent models.

#### 3.2.1 HbO _2_

There were no significant effects of child age (*p*= .113), trial number (*p*= .300), or data type (with versus without SSR,*p*= .228). Adding a term for experimental condition significantly improved model fit (improvement over*m0*: X^2^(2) = 55.18,*p*< .001). Within the final best fit model—*m1:*WTC ~ condition + (1|id)—effects of both full collaboration and collaboration with screen conditions were significant and positive (collaboration: ß = 4.77 x 10^-3^,*SE*= 8.19 x 10^-4^,*p*< .001; collaboration with screen: ß = 5.68 x 10^-3^,*SE*= 8.23 x 10^-4^,*p*< .001, seeTable S4). To identify channel pairs driving the significant main effects, one-sided, paired Welch’s*t*-tests were conducted across condition comparisons with FDR correction for multiple comparisons. Significant differences are represented visually inFigure 5, statistical test results can be found inTable S7, and descriptive statistics are presented inTable 1. For ROI-wise analyses, adding a predictor of age but not of trial (*p*= .561) nor data type (with versus without SSR,*p*= .685) significantly improved the model (age improvement over*m0*: X^2^(1) = 4.53,*p*= .033). There was no effect of condition (*p*= .064). No further post hoc analyses on the specific contribution of ROI-wise comparisons was conducted.

**Fig. 5.**
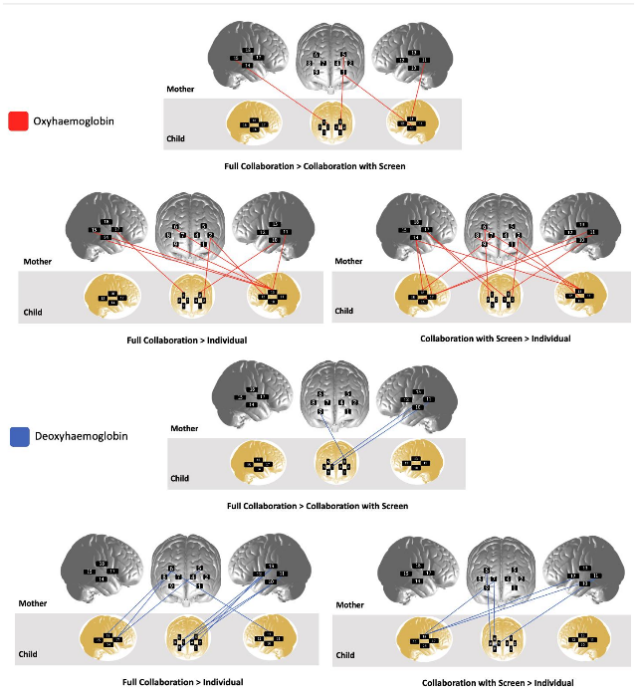
Depiction of significant channel-wise condition comparisons. Channel locations depicted for visualisation purposes only. Short separation channels not represented. For details about optode and channel location, seeTable S1. A list of significant channels and statistics is reported inTable S7. All FDR-corrected*p*< .05.

**Table 1.** Descriptive statistics for neural coherence and task performance.

		Individual		Collaboration with screen		Full collaboration	
		M (±SD)	Min– max	M(±SD)	Min–max	M (±SD)	Min–max
Neural synchrony	Oxyhaemoglobin (HbO _2_ )						
	Trial 1	0.31 (±0.07)	0.12–0.81	0.32 (±0.08)	0.12–0.72	0.32 (±0.07)	0.11–0.72
	Trial 2	0.31 (±0.07)	0.10–0.70	0.32 (±0.07)	0.11–0.72	0.32 (±0.07)	0.13–0.72
	Trial 3	0.31 (±0.07)	0.10–0.74	0.32 (±0.08)	0.12–0.70	0.32 (±0.07)	0.11–0.69
	Total	0.31 (±0.07)	0.10–0.81	0.32 (±0.08)	0.11–0.72	0.32 (±0.07)	0.11–0.72
	Deoxyhaemoglobin (HbR)						
	Trial 1	0.31 (±0.08)	0.12–0.65	0.31 (±0.07)	0.10–0.69	0.31 (±0.08)	0.11–0.75
	Trial 2	0.31 (±0.08)	0.13–0.75	0.31 (±0.07)	0.12–0.72	0.32 (±0.08)	0.12–0.70
	Trial 3	0.32 (±0.08)	0.12–0.75	0.31 (±0.08)	0.11–0.70	0.32 (±0.07)	0.13–0.72
	Total	0.31 (±0.08)	0.12–0.75	0.31 (±0.07)	0.10–0.72	0.31 (±0.08)	0.11–0.75
Task performance	Number puzzles correct						
	Trial 1	0.83 (±1.00)	0.00–3.00	1.92 (±0.70)	1.00–4.00	1.92 (±0.77)	0.00–3.00
	Trial 2	1.31 (±1.31)	0.00–6.00	2.08 (±0.77)	1.00–4.00	2.06 (±0.72)	1.00–3.00
	Trial 3	1.14 (±1.29)	0.00–5.00	2.25 (±0.97)	1.00–5.00	2.14 (±0.90)	1.00–4.00
	Number given						
	Trial 1	1.89 (±0.67)	1.00–3.00	1.97 (±0.74)	1.00–4.00	1.97 (±0.70)	1.00–3.00
	Trial 2	2.11 (±1.04)	1.00–6.00	2.14 (±0.72)	1.00–4.00	2.11 (±0.71)	1.00–3.00
	Trial 3	2.19 (±1.17)	1.00–6.00	2.31 (±0.92)	1.00–5.00	2.14 (±0.90)	1.00–4.00

*Note*. Neural synchrony calculated as all-channel average of WTC. Individual task performance statistics reflect children’s performance.

#### 3.2.2 HbR

Neither age (*p*= .228) nor data type (with versus without SSR,*p*= .150) significantly improved the model. There was a significant effect of trial (improvement over*m0*: X^2^(2) = 9.53,*p*= .009) and of experimental condition (improvement over*m1*: X^2^(2) = 9.17,*p*= .010). The final best fit model,*m2*: WTC ~ trial + condition + (1|id), revealed a significant and positive effect of collaboration condition (ß = 2.51 x 10^-3^,*SE*= 8.30 x 10^-4^,*p*= .002) but not of collaboration with screen condition (*p*= .156, seeTable S5). To identify channel pairs driving main effects of condition, post hoc, one-sided, paired Welch’s*t*-tests were conducted (seeTable 1for descriptive statistics andTable S7for statistical results). For ROI-wise data, no parameter additions improved model fit over the null (addition of trial:*p*= .221, addition of age:*p*= .115, addition of data type (with versus without SSR):*p*= .952; addition of condition:*p*= .512). No post hoc analyses was conducted.

### 3.3 Aim 3: coherence with and without possibility of facial information

Coherence differences in collaboration conditions—*with*(full collaboration) versus*without*(collaboration with screen) information about the interacting partners’ faces—were tested in channel-wise data. In both chromophores, the null model was not significantly improved by the addition of age (HbO_2_:*p*= .122; HbR:*p*= .112), trial number (HbO_2_:*p*= .114; HbR:*p*= .728), data type (with versus without SSR, HbO_2_:*p*= .371; HbR:*p*= .337), nor condition (HbO_2_:*p*= .296; HbR:*p*= .079). This suggests there were no significant differences in average coherence in collaboration conditions with versus without the possibility of referring to each other’s faces.

### 3.4 Aim 4: coherence in true versus pseudodyads

For each condition, in both the channel-wise and ROI-wise analyses, coherence values obtained in the true dyads (*n*= 38 with SSR,*n*= 9 without SSR) were compared against the pseudodyad distributions generated with the phase scrambling method. First, coherence values from all possible long separation channel pairs between children and mothers were averaged to obtain whole-brain coherence values for each dyad. Comparisons were performed using one-sided, paired Welch’s*t*-tests, and*p*-values were FDR corrected for multiple comparisons. Results showed that in both HbO_2_and HbR, true dyad coherence strength was significantly higher than pseudodyad coherence strength in full collaboration and collaboration screen conditions (seeTable 2). In ROI-wise data for both chromophores, coherence in temporal regions was significantly higher in true dyads than in pseudodyads during collaboration conditions (seeFig. 6andTable S9). In channel-wise data, no significant results survived correction for multiple comparisons, but uncorrected results are reported inTable S8.

**Fig. 6.**
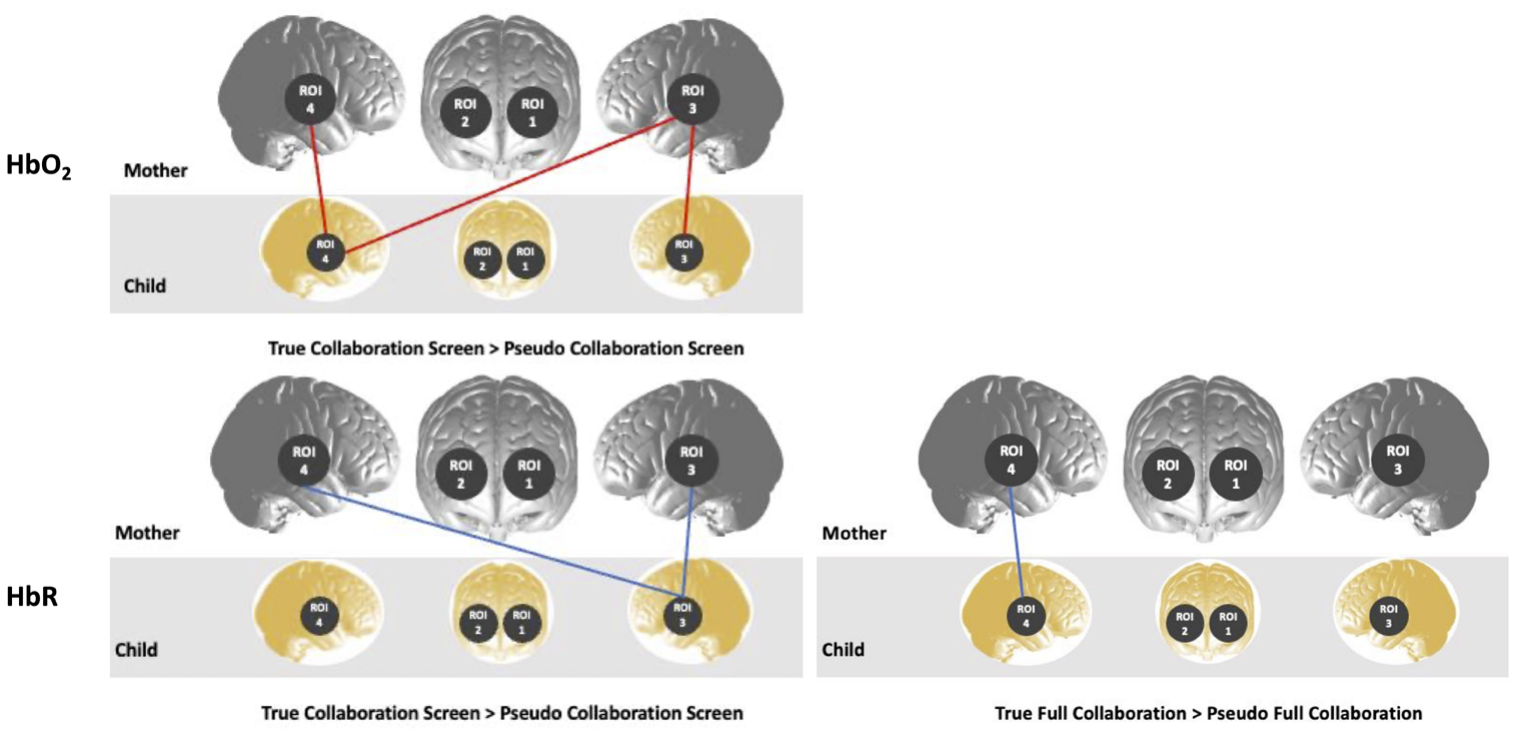
Depiction of ROI pairs with higher synchrony in true versus pseudodyads. Results of one-way, paired Welch’s*t*-tests between*N*= 47 dyads’ true versus generated pseudodyad coherence values in ROI for both chromophores. Top panel represents oxyhaemoglobin and bottom represents deoxyhaemoglobin. All FDR-corrected*p*< .05. Region of interest labels: ROI 1 = left PFC, ROI 2 = right PFC, ROI 3 = left TPJ, ROI 4 = right TPJ.

**Table 2.** Comparison between whole-brain coherence averages in true versus pseudodyads.

Condition	Oxyhaemoglobin (HbO _2_ )	Deoxyhaemoglobin (HbR)
Full collaboration	*t* (46) = 2.43, * p _adj_* = .014*	*t* (46) = 2.01, * p _adj_* *=* .038*
Collaboration screen	*t* (46) = 2.81, * p _adj_* = .011*	*t* (46) = 2.53, * p _adj_* = .038*
Individual	*t* (46) = -0.98, * p _adj_* = .835	*t* (46) = -0.12, * p _adj_* = .379

*Note*. Results of one-way, paired Welch’s*t*-tests between*N*= 47 dyads’ true versus generated pseudodyad coherence values by condition. *p< .05.*p*-values FDR corrected for multiple comparisons.

### 3.5 Aim 5a: coherence strength and collaborative task performance

#### 3.5.1 Task performance

Task performance was measured as the number of puzzles completed correctly by the dyad, and pace of puzzle solving as the number of puzzles the experimenter gave the dyad altogether. Models were built for channel-wise data for HbO_2_and HbR and tested against the best fitting model above (*m1*: WTC ~ condition + (1|id)) for both chromophores.

#### 3.5.2 HbO _2_

The interaction term for number of puzzles completed correctly but not a main effect of pace of problem solving (*p*= .202) significantly contributed to model fit (improvement over*m1*: X^2^(3) = 59.33,*p*< .001). The best fitting model was*m2:*WTC ~ condition*puzzles correct + (1|id) (seeTable S10). Within*m2,*the interaction of full collaboration and number of puzzles solved correctly was significant and positive (ß = 6.74 x 10^-3^,*SE*= 1.22 x 10^-3^,*p*< .001), as was the interaction of collaboration with screen and number of puzzles solved correctly (ß = 8.06 x 10^-3^,*SE*= 1.12 x 10^-3^,*p*< .001).

#### 3.5.3 HbR

The model was improved by the addition of an interaction term for task performance (improvement over*m1*: X^2^(3) = 31.16,*p*< .001) and of a main effect of pace of problem solving (improvement over*m2*: X^2^(1) = 15.21,*p*< .001). Within the final best fit model,*m3:*WTC ~ condition*puzzles correct + (1|id), the interaction of collaboration with screen and number of puzzles correct was significant and positive (ß = 3.53 x 10^-3^,*SE*= 1.22 x 10^-3^,*p*= .004). The interaction of full collaboration and number of puzzles correct was non-significant (*p*= .848, seeTable S11). Descriptive statistics for task performance in all conditions are reported inTable 1.

### 3.6 Aim 5b: coherence strength and background maternal stress

Maternal stress was calculated as sum of the Parental Stress Scale, which estimates general stress levels parents experience (Berry & Jones, 1995). Three outliers fell more than 2 standard deviations above or below the mean and were removed for analysis. Maternal stress was normally distributed (*W*= 0.968,*p*= .178,*M*= 39.18,*SD*= 6.25,*min*= 29,*max*= 52, possible scores between 18 – 90). Models with terms for maternal stress were tested against*m1:*WTC ~ condition + (1|id) for both HbO_2_and HbR.

#### 3.6.1 HbO _2_

An interaction term of condition and maternal stress significantly improved the model (improvement over*m1*: X^2^(3) = 18.18,*p*< .001). There was a significant and negative interaction of maternal stress and full collaboration (ß = -5.24 x 10^-4^,*SE*= 1.47 x 10^-4^,*p*< .001) and of maternal stress and collaboration with screen (ß = -5.55 x 10^-4^,*SE*= 1.47 x 10^-4^,*p*< .001, seeTable S12).

#### 3.6.2 HbR

An interaction for condition and maternal stress significantly improved model fit (improvement over*m1*: X^2^(3) = 26.02,*p*< .001). The interaction of full collaboration and maternal stress was significant and positive (ß = 4.09 x 10^-4^,*SE*= 1.50 x 10^-4^,*p*= .006), while the interaction of collaboration with screen and maternal stress was significant and negative (ß = -3.52 x 10^-4^,*SE*= 1.50 x 10^-4^,*p*= .019, seeTable S13).

## 4 Discussion

This study employed a novel analytical pipeline to developmental fNIRS hyperscanning data to investigate the alignment of functional brain activity of collaborating 4- to 6-year-old children and mothers, over-and-above confounding signals from global physiology and task-related components. Results showed that coherence in cortical haemodynamic activity was significantly stronger during collaborative than during individual problem-solving conditions and that strength of alignment was related specifically to the temporal dynamics of interaction. Neural coherence was not explained solely by the coupling of non-neural physiological rhythms, but the impact of SSR suggested coherence measurements may be artificially inflated by presence of extracerebral haemodynamic signals inevitably captured by fNIRS. Relationships between coherence and behavioural variables were mixed: task performance during full collaboration was positively related to coherence in HbO_2_but not in HbR. Self-reported measures of maternal stress were also significantly related to coherence during full collaboration, in a negative direction in HbO_2_and in a positive direction in HbR. There were no differences in coherence in collaborative conditions with and without the possibility of participants looking to each other’s faces. Overall, coherence between children and mothers during naturalistic collaboration appears to be a robust effect that survives increasingly conservative analysis steps.

### 4.1 Aim 1: reduction of physiological noise

The minimisation of extracerebral noise had a significant dampening effect on coherence strength, particularly in HbO_2_(seeFig. 4,Table S2, andTable S3). In all experimental conditions, average HbO_2_coherence was higher before SSR than after. This aligns with previous research that suggests HbO_2_is impacted by changes in participants’ global physiological processes, such as cardiac oscillations, which are particularly evident in local blood flow changes in the extracerebral layer (Kirilina et al., 2012;Tachtsidis & Scholkmann, 2016). SSR also significantly reduced coherence strength in full collaboration and collaboration with screen conditions in HbR but did not have a significant impact on coherence in the individual condition. During full collaboration, participants could speak with each other which could lead to autonomic responses, and they could engage in postural movement to reach across the table, potentially contributing to changes in blood pressure. The stronger impact of physiological noise on HbO_2_than HbR is expected, as arterioles transporting oxygenated blood away from the heart and into active tissue are thought to be enervated more strongly by the sympathetic nervous system than the venules transporting deoxygenated blood back to the heart (Tachtsidis & Scholkmann, 2016). Some research focuses predominantly on HbR coherence because it is less affected by noise in the extracerebral layer and is more reliably related to fMRI measurements than HbO_2_(Hirsch et al., 2017,^2018^,^2021^).

Previous work with children has also shown it is beneficial to apply SSR to fNIRS data, particularly in naturalistic settings, and that there is very minor to no difference between global versus local SSR when the number of SSCs is small (e.g., four,Pinti et al., 2024). Here, using two SSCs, we have regressed out the average of the available SSCs as we did not expect differences between the two SSR methods. Further, prior work with adults has shown that scalp interference can be heterogeneous across the scalp. Future research using a higher number of SSCs covering different regions of the head might aim to investigate the impact of local regression on reduction of superficial contamination as well as provide a better estimation of the global component. Future work with higher density fNIRS systems will be important to further investigate the heterogeneity of scalp interference in developmental populations alongside the most appropriate regression methods.

Overall, the results presented here indicate that measurement of haemodynamic signals during children’s naturalistic interaction may be artificially inflated by physiological components. Particularly because face-to-face interaction facilitates alignment of physiological rhythms such as heart rate (McCraty, 2017) and respiration patterns (Konvalinka et al., 2022), future research should endeavour to reduce the impact of systemic physiology on measurement of neural coherence. Reporting results in both chromophores, or only in HbR, may also contribute to improvements in the reliability of fNIRS hyperscanning effects in developmental contexts.

### 4.2 Aim 2: coherence in full collaboration compared with individual problem solving

After SSR, neural coherence in both HbO_2_and HbR was stronger in the full collaboration than in the individual condition (seeTable S4andTable S5). These results align with previous research suggesting collaborative tasks may facilitate neural coherence between interacting adults (Czeszumski et al., 2022;Lotter et al., 2023), as well as between parents interacting with children between the ages of 8 and 12 years (Miller et al., 2019), 5 and 9 years (Reindl et al., 2018), and 5 and 6 years of age (Nguyen et al., 2020;Nguyen, Schleihauf, et al., 2021). The present study builds on previous results by including slightly younger children aged 4 to 6 years and by suggesting that coherence effects between children and parents persist after reduction of physiological noise contamination from the extracerebral layer. Building on previous guidelines (e.g.,Nguyen, Hoehl, et al., 2021), incorporating short-separation channels for parent-child fNIRS hyperscanning can improve confidence that signals analysed reflect synchronous states of functional brain activity.

There are many possible explanations for stronger coherence during collaborative than during individual problem solving. Across both chromophores, the majority of significant channel-wise results (collaboration > individual) involved at least one of the participants’ middle frontal gyrus (15 of 18 pairs). fMRI research with adults has suggested the middle frontal gyrus is likely involved in auditory processing (Gernsbacher & Kaschak, 2003;A. L. Giraud et al., 2004), face processing (Sato et al., 2012), and observation of others’ actions (Rizzolatti et al., 1995). It likely also underlies reorientation of attention from exogenous cues to endogenous cues to accomplish certain goals (Japee et al., 2015). The supramarginal gyrus was also implicated in half of all significant channel pairs (9 of 18 pairs). The supramarginal gyrus supports action representation, such as one’s ability to judge the correct hand position to manipulate tools (Lesourd et al., 2017) and to plan their actions to grasp them (Potok et al., 2019). Four of 18 pairs involved a channel over the angular gyrus, which could reflect processes related to language, memory, visuospatial attention, number processing, motor planning, and body awareness (Wagner & Rusconi, 2022). An opinion piece byRedcay and Schilbach (2019)also highlighted the role of the temporoparietal junction in supporting the mentalising network more broadly. It may be that synchrony in the functional activity of these regions reflects the alignment of interacting individuals’ communicative and social cognitive processes, as well as mutual prediction and action representation (see alsoHoehl et al., 2021;Redcay & Schilbach, 2019). However, the current study was not designed to localise effects, and the results presented here cannot provide direct evidence for a specific interpretation. Overall, the results suggest that brain-to-brain coherence specific to the coordinated temporal dynamics of face-to-face collaboration emerges in prefrontal and temporal regions between 4- to 6-year-old children collaborating with their mothers.

It is interesting that channel pairs which showed stronger synchrony in collaboration than individual conditions involved channels within the same regions (e.g., homologous) and across different regions (e.g., heterologous). It might be that these two categories are functionally different from each other.Jiang et al. (2021)argue for two types of interpersonal neural synchrony: one that arises from common external input, like auditory processing, which may be reflected in homologous brain regions, and another that arises from interactive processes, such as one partner speaking and the other listening, which could result in heterologous pairs. We could speculate that the homologous channel pairs here reflect neural synchrony elicited by similar perceptual or cognitive processes, such as planning similar steps to solve the Tangram puzzle. Heterologous pairs, on the other hand, could reflect one’s attunement to the state of the other person as they listen to what they say or watch how they move the puzzle pieces. Future research might test this distinction directly.

Another point of convergence between this study and those previous is the lack of robust condition-based difference in coherence when signals are averaged within regions of interest. In this study, the best fit model for HbO_2_did reveal a main effect of condition, driven a positive effect of the collaboration with screen. In post hoc*t*-tests, no region of interest condition comparisons survived correction for multiple comparisons. No parameter additions over the null improved model fit for ROI-wise HbR coherence. Similarly, neitherMiller et al. (2019)norNguyen et al. (2020)found significant main effects in their primary coherence analysis of ROI-wise data. It may be that neural coherence detected at the level of channel-wise observations is distributed across regions of interest. One fNIRS study reported that averaging more than two channel pairs’ interhemispheric coherence signal decreased statistical sensitivity to detect differences between groups that were present in unaggregated data (Oni et al., 2023). It seems likely that individual channels, even within the same region of interest, may capture unique variability in the haemodynamic signal that is then obscured by averaging signals within ROI. ROI analyses are useful to handle large amounts of data, but they may make a lack of significant differences difficult to interpret.

### 4.3 Aim 3: coherence with and without possibility of facial information

This study examined coherence strength in two collaborative conditions: full collaboration, where participants could see each other as they would in a typical interaction, and collaboration with screen, where a half-screen was drawn between participants. In the collaboration with screen condition, participants could still talk and see each other’s hands as they worked together to solve a puzzle, but they could not see each other’s faces. Previous research with adults suggests that the strength of brain-to-brain synchrony relates to patterns of direct eye contact (Dravida et al, 2020;Hirsch et al., 2017;Noah et al., 2020;Saito et al., 2010). In this study, comparison of synchrony strength in full collaboration and collaboration with screen conditions did not reveal robust differences in coherence in either HbO_2_or HbR. From a functional perspective, this suggests that coherence during full collaboration cannot be explained solely by the coordination of eye gaze or, more broadly, by cues available on the face, such as one’s emotional state or their visual speech information.

The lack of effect of a partial screen on strength of coherence does not refute previous work suggesting eye gaze relates to neural synchrony but rather builds on it. Adult fNIRS hyperscanning research has shown coherence in both HbO_2_(Noah et al., 2020) and HbR (Hirsch et al., 2017) in prefrontal and temporoparietal regions is stronger during eye-to-eye contact than when participants looked at faces in picture (Hirsch et al., 2017) and video (Noah et al., 2020) stimuli. While synchrony strength may be stronger in live eye contact than when looking to eyes in a picture or dynamic video, it may also be the case that direct eye contact is not a necessary pre-requisite for alignment of functional brain activity in social interaction.Haresign et al. (2023)found that interbrain synchrony measured with EEG was not related to instances of infant–caregiver mutual gaze, which could indicate that mutual gaze enhances and/or maintains previously established states of neural synchrony, rather than elicits it directly. A similar interpretation could be offered here: coherence can emerge in the absence of information from the eyes and face, perhaps just as strongly as when face information is available. When participants cannot rely on information from their partner’s eyes and faces, such as their gaze direction or emotional states, they might rely on compensatory or alternative strategies that also give rise to coherence. Some suggest that manual manipulation of objects in the shared environment may give rise to states of shared attention (Sun et al., 2024;Yu & Smith, 2017), particularly among older children (Franchak et al., 2024). In this view, information from each other’s faces may not be necessary, as children take turns with their parents moving puzzle pieces, indicating or gesturing to each other, and discussing, planning, and implementing their next steps. Future research may investigate whether the strength of synchronous neural states is modulated by manipulation of salient objects, manual behaviours such as pointing, gesturing, or demonstrating, looks to each other’s hands, or the timing or content of verbal exchanges.

### 4.4 Aim 4: coherence in true versus pseudodyads

Phase-scrambled pseudodyad analyses were conducted to rule out the explanation that synchrony emerged only because children and their parents were performing the same task in the same environment. Surrogate data were generated such that each mother’s fNIRS signal was phase scrambled (Simony et al., 2016) 100 times and WTC with their child’s signal was calculated and averaged across the iterations. This procedure preserved the original signal’s frequency, which is thought to relate to the task and the environment, while disrupting any meaningful temporal information related to coordinated social interaction. Comparison of true versus pseudodyad coherence revealed that, at the whole-brain level, synchrony in both HbO_2_and HbR during collaboration was significantly stronger among true dyads than among pseudodyads. This suggests that changes in alignment of dyads’ haemodynamic activity were stronger in signals that retained the original temporal dimension of the mother and child’s real interaction compared with surrogate data where the mothers’ signal reflected only task-related components. There were no differences in coherence strength between true versus pseudodyads in the individual condition in either chromophore. At the whole-brain level, both HbO_2_and HbR coherence seems to reflect temporal dynamics of social interaction during collaborative problem solving, and neither can be explained entirely by bottom-up processing of a shared task or environment.

ROI-wise pseudodyad results in both chromophores suggest that, during collaboration conditions, coherence between participants’ temporal regions was significantly stronger in true dyads than in pseudodyads (seeFig. 6). This is difficult to interpret given that the main ROI-wise analyses revealed no effect of condition in ROI-wise data in either chromophore. It could be that synchrony in prefrontal regions relates to task-dependent processes such as planning and execution, whereas temporal region synchrony is driven primarily by mutual prediction. At the channel level, no significant differences in true versus pseudodyad coherence in either chromophore in any condition survived correction for multiple comparisons. Future research is required to fully extricate the links between the timing of mutually engaged interaction and the alignment of two individuals’ functional brain activity. Overall, coherence in collaboration of true dyads, who engaged in real, coordinated interaction, was significantly higher than in phase-scrambled surrogate data. This supports the interpretation that the brain-to-brain coherence effects reported here cannot be dismissed entirely as an epiphenomenon, and that they are at least partially related to the timing of mutually contingent social interaction.

### 4.5 Aim 5a: coherence strength and collaborative task performance

Coherence during collaboration was positively related to the number of puzzles dyads solved correctly. In HbO_2_, coherence strength was positively related to the number of puzzles solved correctly during full collaboration. This replicates the pattern reported byNguyen et al. (2020)who originally designed and implemented the Tangram puzzle-solving task with a similar age range. The task seems well-suited for capturing meaningful variability in problem-solving performance of 4- to 6-year-old children collaborating with their parents. In the present study, the relationship between HbO_2_coherence was also positively predicted by the number of puzzles solved in the collaboration with screen condition, where participants could not see each other’s faces. This is a novel result that suggests the link between coherence and task performance cannot be mediated entirely by eye contact or the role of facial information during collaboration.

Unlike in HbO_2_, the best fit HbR model included a significant, positive main effect of the number of puzzles given to the dyad. This suggests that dyads who completed puzzles more quickly (across all conditions) also showed stronger coherence in HbR than those who completed puzzles more slowly. The term was included in the model to control for potential effects of pace, and the main interaction of interest (Task Performance x Condition) was added. The final model revealed no interaction of number of puzzles completed correctly during full collaboration. In the collaboration with screen condition, number of puzzles completed correctly predicted significant variance in HbR coherence strength.

The pattern of effects reported here replicates and contributes some new insight into the correlation between neural coherence and collaborative task performance. The causal pathway underlying this relationship remains unknown. It could be that synchrony paves the way for collaborative success, or that well-coordinated collaborative behaviours give rise to coherence, or that, over the course of the interaction, synchrony and collaborative success mutually reinforce one another in a bidirectional relationship. A small number of studies have delivered synchronised transcranial electrical stimulation to dyads of adults, one during a finger-tapping task (Novembre et al., 2017) and one during teaching/learning of songs (Pan et al., 2021). The results suggest that synchronous currents delivery enhanced social behaviours in the form of synchronous tapping (Novembre et al., 2017) and intonation learning (Pan et al., 2021). Future multibrain stimulation studies would be required to determine whether neural synchrony causes or directly enhances coordinated social behaviours, such as successful collaborative problem solving (Novembre & Iannetti, 2020).

### 4.6 Aim 5b: coherence strength and background maternal stress

Background maternal stress predicted significant variance in both HbO_2_and HbR coherence during collaboration. The effects in HbO_2_coherence in full collaboration replicate pattern reported byNguyen et al. (2020)andAzhari et al. (2019), such that mothers who reported higher levels of background stress also showed weaker HbO_2_coherence than those with lower stress levels. A novel relationship is reported here in HbR—higher (than lower) levels of maternal stress were related to stronger HbR coherence during collaboration. While stress may have a dampening effect on the coherence of concentration changes in HbO_2_, it may have an attenuating effect on coherence in HbR. In both chromophores, coherence during the collaboration with screen condition was also negatively related to self-reported background maternal stress levels. It seems reasonable to predict that neural coherence is sensitive to proximal factors such as the background traits of the interacting individuals. Evidence from behavioural research has suggested quality of parent–child interactions may be negatively related to overall family stress (McKay et al., 1996) and chronic physiological stress (Tarullo et al., 2017). One possible explanation for the negative relationship between stress and synchrony is that mothers who experience high levels of background stress may be less able to coordinate and flexibly adapt their social behaviours during interaction. Importantly, some previous research has not replicated the significant relationship between parental stress and strength of neural synchrony during children’s collaborative problem solving (Nguyen, Schleihauf, et al., 2021). Future research is required to clarify the link between coherence and individual-specific factors that may underlie successful collaborative interactions.

## 5 Conclusion

This work sits at the intersection of related scientific efforts: that of the fNIRS field to standardise analytical decisions and their reporting to ensure proper interpretation of optical neuroimaging data (Yücel et al., 2021) and, in the field of two-person neuroscience, the need for clear theoretical explanations to account for neural coherence (Holroyd, 2022).Holroyd (2022)argues that the field of interbrain synchrony is preparing for a replication crisis (Open Science Collaboration, 2015), as vague definitions of neural synchrony persist alongside rapid methodological developments that continue to fuel new research. One proposed remedy to this problem is “to do exactly what the name implies: replicate” (Holroyd, 2022). The present study is a direct attempt to do so while applying best practices in the analytical treatment of functional brain imaging data acquired with optical methods during a naturalistic hyperscanning task. The results replicate previous findings that coherence emerges more strongly in collaborative than in individual problem solving between 4- to 6-year-old children and their mothers, but it extends current understanding to suggest that (i) the presence of systemic physiology measured in the extracerebral layer may artificially inflate measures of brain-to-brain synchrony, particularly in oxygenated haemoglobin and (ii) coherence during collaboration cannot be explained entirely by the alignment of task- or environmental-related frequency components in the fNIRS signal but is at least partially related to the dynamics of real-time social interaction.

## Supplementary Material

Supplementary Material

## Data Availability

The data supporting the results of this paper may be made available upon reasonable request to the corresponding author through a formal data sharing and project affiliation agreement. Code has been published publicly at the following link:https://github.com/vmousley/pc_fNIRS_hyperscanning.
