# Supplementary material for: Analytical pipeline optimisation in developmental fNIRS hyperscanning data: Neural coherence between 4- to 6-year old children collaborating with their mothers
Source: Imaging Neurosci (Camb). 2025 Mar 20;3:imag_a_00509. doi: 10.1162/imag_a_00509 (PMC12319887; doi:10.1162/imag_a_00509)
Supplement: Supplementary Material [file imag_a_00509-supp.pdf]

Supplementary Materials

Table S1: Long separation channel MNI coordinates and LPBA labels.

Chan #	*MNI Coordinates* x	y	z	*LPBA Label (% Overlap)*	*Number included* Children	Mothers
1	-33	63	14	L middle frontal gyrus (100.00%)	43	48
2	-39	50	28	L middle frontal gyrus (100.00%)	29	28
*3**	-41	56	19	L middle frontal gyrus (99.19%)	16	32
4	-20	67	24	L middle frontal gyrus (76.58%)	35	44
	-	-	-	L superior frontal gyrus (23.42%)	-	-
5	-25	54	36	L middle frontal gyrus (100.00%)	17	27
6	27	55	36	R middle frontal gyrus (99.13%)	20	19
7	20	68	24	R middle frontal gyrus (96.00%)	40	40
8	33	65	14	R middle frontal gyrus (100.00%)	37	31
9	41	51	28	R middle frontal gyrus (90.99%)	42	46
10	-70	-44	6	L middle temporal gyrus (61.30%)	24	32
	-	-	-	L superior temporal gyrus (38.70%)	-	-
11	-63	-58	26	L angular gyrus (79.38%)	27	31
12	-68	-22	29	L supramarginal gyrus (76.01%)	38	31
13	-64	-38	46	L supramarginal gyrus (97.20%)	36	28
14	69	-51	-4	R middle temporal gyrus (86.76%)	28	19
15	60	-67	13	R middle occipital gyrus (62.41%)	26	20
*16**	62	-65	-4	R inferior temporal gyrus (50.18%)	10	24
	-	-	-	R middle occipital gyrus (26.69%)	-	-
	-	-	-	R middle temporal gyrus (23.13%)	-	-
17	72	-31	20	R superior temporal gyrus (60.56%)	37	38
	-	-	-	R supramarginal gyrus (30.12%)	-	-
18	66	-47	37	R angular gyrus (68.43%)	40	34
	-	-	-	R supramarginal gyrus (29.23%)	-	-

Note. Asterisks (*) indicate short separation channels which were excluded for analysis. LPBA = LONI Probabilistic Brain Atlas. Number of valid channels calculated at participant level before two dyads were excluded for failure to reach inclusion criteria of at least one valid region per participant in the dyad (*N* = 49 dyads).

Table S2: Impact of physiological noise reduction per chromophore by condition.

**Condition**	**Oxyhaemoglobin (HbO_2)**	**Deoxyhaemoglobin (HbR)**
FullCollaboration	$t(4877) = 4.83$, $p_{adj} < .001$ ***	$t(4877) = 3.51$, $p_{adj} < .001$ ***
CollaborationScreen	$t(4877) = 6.84$, $p_{adj} < .001$ ***	$t(4877) = 2.91$, $p_{adj} = .003$ **
Individual	$t(4877) = 6.61$, $p_{adj} < .001$ ***	$t(4877) = 0.82$, $p_{adj} = .207$

Note. Tests performed were Welch's one-sided, paired *t*-tests from $n = 38$ dyads who contributed fNIRS data both with and without SSR. Regression required at least one valid short separation channel per dyad. *P*-values FDR-corrected. Units = Micromolar per litre. *** $p < .001$, ** $p < .01$, * $p < .05$.

Table S3: Descriptive statistics of physiological noise reduction impact.

Condition	Oxyhaemoglobin (HbO_2)		Deoxyhaemoglobin (HbR)	
	M (SD)	***Min - Max***	***M (SD)***	***Min - Max***
FullCollaboration	2.50×10^3 (-9.21×10^{-5})	1.49×10^3 – 5.51×10^2	1.34×10^3 (6.58×10^4)	2.36×10^2 – 3.22×10^2
CollaborationScreen	3.62×10^3 (2.43×10^3)	1.15×10^2 – 2.01×10^2	1.09×10^3 (9.44×10^4)	-4.94×10^3 – 1.28×10^2
Individual	3.61×10^3 (2.35×10^3)	2.40×10^2 – 2.22×10^2	3.08×10^4 (9.08×10^4)	-1.29×10^2 – 2.02×10^2

Note. All values calculated as difference between value derived from without SSR minus with SSR data. Units = Micromolar per litre.

Table S4: HbO_2 coherence by condition (channel-wise).

**Fixed Effects**						
	Estimate	*SE*	*95% CI*	*df*	*t*	*p*
Intercept	0.31	1.64×10^{-3}	[0.31, 0.31]	48521	190.55	< .001 ***
FullCollaboration	4.77×10^{-3}	8.19×10^{-4}	[3.17×10^{-3}, 6.38×10^{-3}]	48521	5.83	< .001 ***
CollaborationScreen	5.68×10^{-3}	8.23×10^{-4}	[4.07×10^{-3}, 7.29×10^{-3}]	48521	6.91	< .001 ***
**Random Effects**						
		Variance			*SD*	
Participant (Intercept)		1.08×10^{-4}			0.01	
**Model Fit**						
		Marginal			*Conditional*	
R^2		1.11×10^{-3}			0.02	
REML criterion		- 115099.00				

Note. Model: WTC ~ condition + (1|id). *P*-values for fixed effects calculated using Satterthwaite's method, and confidence intervals and *p*-values computed used a Wald *t*-distribution approximation. *** $p < .001$, ** $p < .01$, * $p < .05$.

Table S5: HbR coherence by condition (channel-wise).

**Fixed Effects**						
	Estimate	*SE*	*95% CI*	*df*	*t*	*p*
Intercept	0.31	1.49×10^{-3}	[0.31, 0.32]	48519	209.88	< .001 ***
Trial 2	-2.41×10^{-3}	8.21×10^{-4}	[-4.02×10^{-3}, -8.02×10^{-4}]	48519	-2.94	.003 **
Trial 3	-5.30×10^{-4}	8.43×10^{-4}	[-2.18×10^{-3}, 1.12×10^{-3}]	48519	-0.63	.530
FullCollaboration	2.51×10^{-3}	8.30×10^{-4}	[8.85×10^{-4}, 4.14×10^{-3}]	48519	3.03	.002 **
CollaborationScreen	1.18×10^{-3}	8.34×10^{-4}	[-4.50×10^{-4}, 2.82×10^{-3}]	48519	1.42	.156
**Random Effects**						
		Variance			*SD*	
Participant (Intercept)		7.60×10^{-5}			8.72×10^{-3}	
**Model Fit**						
		Marginal			*Conditional*	
R^2		3.80×10^{-4}			0.01	

REML criterion	- 113794.30

Note. Model: WTC ~ trial + condition + (1|id). *P*-values for fixed effects calculated using Satterthwaite's method, and confidence intervals and *p*-values computed used a Wald *t*-distribution approximation. *** $p < .001$, ** $p < .01$, * $p < .05$.

Table S6: Likelihood Ratio Test results for condition effects in data with and without superficial signal regression in both chromophores.

**HbO_2: Without SSR**							
Model	*N Parameters*	*AIC*	*BIC*	*Deviance*	*Statistic*	*DF*	*P-value*
0	3	-114678	-114652	-114684	-	-	-
1	5	-114722	-114678	-114732	48.11	2	< .001 ***
**HbO_2: With SSR**							
Model	*N Parameters*	*AIC*	*BIC*	*Deviance*	*Statistic*	*DF*	*P-value*
0	3	-101078	-101052	-101084	-	-	-
1	5	-101124	-101081	-101134	49.95	2	< .001 ***
**HbR: Without SSR**							
Model	*N Parameters*	*AIC*	*BIC*	*Deviance*	*Statistic*	*DF*	*P-value*
0	3	-113290	-113183	-113215	-	-	-
1	5	-113223	-113179	-113233	17.89	2	.001 **
**HbR: With SSR**							
Model	*N Parameters*	*AIC*	*BIC*	*Deviance*	*Statistic*	*DF*	*P-value*
0	3	-99805	-99779	-99811	-	-	-
1	5	-99822	-99779	-99832	21.66	2	< .001 ***

Note. m0: WTC ~ (1|id), m1: WTC ~ condition + (1|id). A *p*-value < 0.05 indicates significant model improvement (i.e., better explanatory power) compared to the base model.

Table S7: Significant results of channel-wise Welch's *t*-tests.

**Condition Comparison**	**Oxyhaemoglobin (HbO_2)**	**Deoxyhaemoglobin (HbR)**
FullCollaboration > CollaborationScreen	C1 – M5 ($p_{adj} = .048$) *	C5 – M9 ($p_{adj} = .037$) *
	C7 – M15 ($p_{adj} = .013$) *	C7 – M10 ($p_{adj} = .013$) *
	C10 – M1 ($p_{adj} = .034$) *	C9 – M11 ($p_{adj} = .047$) *
	C13 – M11 ($p_{adj} = .016$) *	-
FullCollaboration > Individual	C2 – M2 ($p_{adj} = .018$) *	C2 – M12 ($p_{adj} = .004$) **
	C5 – M10 ($p_{adj} = .031$) *	C7 – M10 ($p_{adj} = .045$) *
	C6 – M17 ($p_{adj} = .028$) *	C7 – M13 ($p_{adj} = .033$) *
	C12 – M2 ($p_{adj} = .003$) **	C9 – M10 ($p_{adj} = .005$) **
	C13 – M6 ($p_{adj} = .027$) *	C9 – M11 ($p_{adj} = .005$) *
	C13 – M9 ($p_{adj} = .022$) *	C13 – M6 ($p_{adj} = 047$) *
	C13 – M11 ($p_{adj} = .016$) *	C17 – M5 ($p_{adj} = .022$) *
	C13 – M14 ($p_{adj} = .028$) *	C17 – M6 ($p_{adj} = .006$) **
	C13 – M15 ($p_{adj} = .023$) *	C18 – M6 ($p_{adj} = .041$) *
CollaborationScreen > Individual	C2 – M2 ($p_{adj} = .014$) *	C8 – M6 ($p_{adj} = .001$) **
	C4 – M17 ($p_{adj} = .047$) *	C9 – M7 ($p_{adj} = .047$) *
	C5 – M10 ($p_{adj} = .028$) *	C9 – M11 ($p_{adj} = .047$) *
	C6 – M17 ($p_{adj} = .038$) *	C18 – M7 ($p_{adj} = .024$) *
	C8 – M6 ($p_{adj} = 038$) *	C18 – M11 ($p_{adj} = .022$) *
	C10 – M2 ($p_{adj} = .007$) **	C18 – M12 ($p_{adj} = .049$) *
	C13 – M6 ($p_{adj} = .041$) *	-

	C13 – M8 ($p_{adj} = .018$) *	-
	C13 – M14 ($p_{adj} = .028$) *	-
	C14 – M11 ($p_{adj} = .038$) *	-
	C17 – M11 ($p_{adj} = .010$) *	-
	C17 – M14 ($p_{adj} = .029$) *	-
	C18 – M7 ($p_{adj} = .030$) *	-
	C18 – M14 ($p_{adj} = .046$) *	

Note. Significant results of one-sided, paired Welch's *t*-tests of channel pairings for condition comparisons of coherence. First number in channel pairs represents child's channel, second number represents mother's channel. Data analysed for $n = 38$ datasets with SSR and $n = 9$ without SSR. Coherence averaged over trials. *P*-values FDR-corrected. *** $p < .001$, ** $p < .01$, * $p < .05$.

Table S8: Uncorrected, significant differences in coherence in true versus pseudodyads per channel pair by condition.

**Condition Comparison**	**Oxyhaemoglobin (HbO_2)**	**Deoxyhaemoglobin (HbR)**
FullCollaboration	C1 – M5 ($p = .036$) *	C2 – M5 ($p = .045$) *
	C2 – M2 ($p = .011$) *	C4 – M5 ($p = .032$) *
	C6 – M10 ($p = .008$) **	C5 – M10 ($p = .037$) *
	C6 – M14 ($p = .015$) *	C5 – M17 ($p = .033$) *
	C7 – M10 ($p = .047$) *	C7 – M10 ($p = .040$) *
	C7 – M14 ($p = .006$) **	C8 – M14 ($p = .029$) *
	C8 – M10 ($p = .035$) *	C8 – M15 ($p = .016$) *
	C9 – M9 ($p = .006$) **	C8 – M17 ($p = .013$) *
	C10 – M14 ($p = .010$) *	C9 – M11 ($p = .020$) *
	C11 – M10 ($p = .022$) *	C9 – M12 ($p = .020$) *
	C11 – M11 ($p = .034$) *	C10 – M5 ($p = .018$) *
	C10 – M10 ($p = .043$) *	C10 – M7 ($p = .029$) *
	C12 – M1 (p = .039) *	C10 – M10 ($p = .001$) **
	C12 – M2 ($p = .021$) *	C10 – M11 ($p = .018$) *
	C12 – M4 (p = .046) *	C10 – M12 ($p = .048$) *
	C12 – M10 ($p = .034$) *	C10 – M13 ($p = .013$) *
	C12 – M14 ($p = .020$) *	C10 – M18 ($p = .023$) *
	C13 – M7 ($p = .001$) **	C11 – M11 ($p = .046$) *
	C13 – M9 ($p = .037$) *	C11 – M18 ($p = .047$) *
	C13 – M11 ($p = .008$) **	C12 – M11 ($p = .013$) *
	C13 – M14 ($p = .014$) *	C12 – M14 ($p = .040$) *
	C13 – C15 ($p = .041$) *	C12 – M17 ($p = .035$) *
	C14 – M14 ($p = .026$) *	C13 – M5 ($p = .015$) *
	C14 – M17 ($p = .005$) **	C13 – M6 ($p = .039$) *
	C17 – M4 ($p = .041$) *	C13 – M10 ($p = .027$) *
	C17 – M9 ($p = .028$) *	C13 – M14 ($p = .018$) *
	C17 – M17 ($p = .008$) **	C13 – M17 ($p = .023$) *
	C18 – M6 ($p = .041$) *	C14 – M12 ($p = .003$) **
		C14 – M15 ($p = .029$) *
		C15 – M10 ($p = .028$) *
		C15 – M12 ($p = .039$) *
		C15 – M18 ($p = .014$) *
		C17 – M2 ($p = .002$) **

		C17 – M5 (p = .028) *<br>C17 – M6 (p = .009) **<br>C17 – M7 (p = .036) *<br>C17 – M14 (p = .010) *<br>C17 – M15 (p = .005) **<br>C17 – M17 (p = .011) *<br>C18 – M6 (p = .013) *<br>C18 – M14 (p = .033) *<br>C18 – M18 (p = .047) *
CollaborationScreen	C1 – M6 (p = .048) *<br>C2 – M7 (p = .019) *<br>C2 – M8 (p = .038) *<br>C2 – M14 (p = .010) *<br>C4 – M10 (p = .022) *<br>C4 – M14 (p = .006) *<br>C4 – M17 (p = .042) *<br>C4 – M18 (p = .009) **<br>C6 – M8 (p = .006) **<br>C7 – M4 (p = .033) *<br>C8 – M4 (p = .033) *<br>C8 – M6 (p = .003) **<br>C8 – M8 (p = .014) *<br>C9 – M14 (p = .028) *<br>C10 – M2 (p = .013) *<br>C10 – M5 (p = .032) *<br>C10 – M8 (p = .037) *<br>C10 – M10 (p = .035) *<br>C10 – M11 (p = .011) *<br>C10 – M14 (p < .001) ***<br>C10 – M17 (p = .003) **<br>C10 – M18 (p = .026) *<br>C11 – M10 (p = .017) *<br>C11 – M11 (p = .007) **<br>C11 – M14 (p = .005) **<br>C11 – M18 (p = .019) *<br>C12 – M4 (p = .014) *<br>C12 – M8 (p = .019) *<br>C12 – M10 (p = .009) **<br>C12 – M11 (p = .011) *<br>C12 – M14 (p = .030) *<br>C12 – M18 (p = .044) *<br>C13 – M6 (p = .017) *<br>C13 – M14 (p = .005) **<br>C13 – M18 (p = .017) *<br>C14 – M11 (p = .025) *<br>C14 – M18 (p = .018) *<br>C15 – M18 (p = .028) *<br>C17 – M11 (p = .012) *<br>C17 – M12 (p = .020) *<br>C17 – M14 (p = .003) **	C1 – M6 (p = .049) *<br>C2 – M14 (p = .005) **<br>C4 – M7 (p = .046) *<br>C5 – M12 (p = .041) *<br>C6 – M4 (p = .025) *<br>C6 – M7 (p = .023) *<br>C8 – M6 (p = .002) **<br>C8 – M18 (p = .022) *<br>C9 – M6 (p = .016) *<br>C10 – M1 (p = .017) *<br>C10 – M4 (p = .038) *<br>C10 – M5 (p = .011) *<br>C10 – M6 (p = .031) *<br>C10 – M9 (p = .014) *<br>C10 – M10 (p = .010) *<br>C10 – M11 (p = .025) *<br>C10 – M12 (p = .026) *<br>C10 – M13 (p = .008) **<br>C10 – M15 (p = .020) *<br>C10 – M17 (p = .004) **<br>C10 – M18 (p = .011) *<br>C11 – M10 (p = .011) *<br>C11 – M12 (p = .024) *<br>C11 – M14 (p = .031) *<br>C12 – M10 (p = .036) *<br>C13 – M10 (p = .041) *<br>C13 – M12 (p = .008) **<br>C13 – M13 (p = .040) *<br>C13 – M15 (p = .041) *<br>C13 – M17 (p = .008) **<br>C13 – M18 (p = .032) *<br>C17 – M9 (p = .049) *<br>C17 – M10 (p = .009) **<br>C17 – M12 (p = .024) *<br>C18 – M1 (p = .022) *<br>C18 – M7 (p = .033) *<br>C18 – M11 (p = .031) *<br>C18 – M12 (p = .041) *<br>C18 – M13 (p = .024) *<br>C18 – M18 (p = .003) **

	C17 – M17 (p = .012) *<br>C17 – M18 (p = .006) **<br>C18 – M1 (p = .004) **<br>C18 – M6 (p = .027) *<br>C18 – M7 (p < .001) ***<br>C18 – M9 (p = .040) *<br>C18 – M11 (p = .021) *<br>C18 – M12 (p = .044) *<br>C18 – M17 (p = .010) *<br>C18 – M18 (p = .004) **	
Individual	C8 – M10 (p = .046) *<br>C8 – M12 (p = .032) *<br>C10 – M6 (p = .046) *<br>C10 – M10 (p = .008) **<br>C10 – M12 (p = .030) *<br>C12 – M10 (p = .019) *<br>C17 – M17 (p = .031) *<br>C18 – M6 (p = .007) **	C10 – M10 (p = .021) *<br>C10 – M11 (p = .010) *<br>C10 – M13 (p = .032) *<br>C10 – M17 (p = .031) *<br>C10 – M18 (p = .012) *<br>C11 – M10 (p = .020) *<br>C11 – M13 (p = .045) *<br>C12 – M17 (p = .024) *<br>C14 – M13 (p = .020) *<br>C15 – M4 (p = .019) *<br>C17 – M12 (p = .037) *<br>C17 – M15 (p = .020) *<br>C18 – M17 (p = .015) *

Note. Significant results of uncorrected, one-sided, paired *t*-tests of channel-wise pairs for true > pseudodyad coherence by condition before FDR-correction for multiple comparisons. No comparison survived correction. First number in channel pairs represents child's channel, second number represents mother's channel. Coherence averaged over trials. *** $p < .001$, ** $p < .01$, * $p < .05$.

Table S9: Significant results of pseudodyad analysis ROI-wise *t*-tests after FDR-correction.

**Condition Comparison**	**Oxyhaemoglobin (HbO_2)**	**Deoxyhaemoglobin (HbR)**
FullCollaboration	-	C4 – M4 (p_{adj} = .045) *
CollaborationScreen	C3 – M4 (p_{adj} = .015) *<br>C4 – M3 (p_{adj} = .045) *<br>C4 – M4 (p_{adj} = .019) *	C3 – M3 (p_{adj} = .013) *<br>C3 – M4 (p_{adj} = .013) *
Individual	-	-

Note. Significant results of one-sided, paired *t*-tests of ROI pairings for true > pseudodyad coherence by condition. First number in ROI pairs represents child's region, second number represents mother's region. ROI 1 = right PFC, ROI 2 = left PFC, ROI 3 = right TPJ, ROI 4 = left TPJ. Coherence averaged over trials. *P*-values FDR-corrected. *** $p < .001$, ** $p < .01$, * $p < .05$.

Table S10: HbO_2 coherence and task performance.

**Fixed Effects**						
	Estimate	*SE*	*95% CI*	*df*	*t*	*p*
Intercept	0.31	$2.19 x 10^{-3}$	[0.31, 0.32]	29927	142.5<br>3	< .001 ***
FullCollaboration	$-7.23 x 10^{-3}$	$2.53 x 10^{-3}$	[-0.01, -<br>$2.28 x 10^{-3}$]	29927	-2.86	.004 **

CollaborationScreen	-5.77x10^{-3}	2.32x10^{-3}	[-0.01, -1.23x10^{-3}]	29927	-2.49	.013 *
NumCorrect	-2.94x10^{-3}	6.20x10^{-4}	[-4.16x10^{-3}, -1.73x10^{-3}]	29927	-4.75	< .001 ***
FullCollaboration x NumCorrect	6.74x10^{-3}	1.22x10^{-3}	[4.35x10^{-3}, 9.13x10^{-3}]	29927	5.53	< .001 ***
CollaborationScreen x NumCorrect	8.06x10^{-3}	1.12x10^{-3}	[5.87x10^{-3}, 0.01]	29927	7.20	< .001 ***
**Random Effects**						
	Variance			*SD*		
Participant (Intercept)	1.01x10^{-4}			0.01		
**Model Fit**						
	Marginal			*Conditional*		
R^2	4.62x10^{-3}			0.02		
REML criterion	- 71214.80					

Note. Model: WTC ~ condition*puzzles correct + (1|id). *P*-values for fixed effects calculated using Satterthwaite's method, and confidence intervals and *p*-values computed used a Wald *t*-distribution approximation. *** $p < .001$, ** $p < .01$, * $p < .05$.

Table S11: HbR coherence and task performance.

**Fixed Effects**						
	Estimate	*SE*	*95% CI*	*df*	*t*	*p*
Intercept	0.03	2.60x10^{-3}	[0.03, 0.03]	29926	117.42	< .001 ***
NumGiven	4.88x10^{-3}	1.25x10^{-3}	[2.42x10^{-3}, 7.33x10^{-3}]	29926	3.90	< .001 ***
FullCollaboration	6.43x10^{-3}	2.96x10^{-3}	[6.39x10^{-4}, 0.01]	29926	2.18	.030 *
CollaborationScreen	-3.75x10^{-3}	2.78x10^{-3}	[-3.75x10^{-3}, 1.70x10^{-3}]	29926	-1.35	.178
NumCorrect	-2.50x10^{-3}	9.73x10^{-4}	[-4.40x10^{-3}, -5.87x10^{-4}]	29926	-2.56	.010 *
FullCollaboration x NumCorrect	-1.28x10^{-3}	1.31x10^{-3}	[-3.85x10^{-3}, 1.29x10^{-3}]	29926	-0.97	.330
CollaborationScreen x NumCorrect	3.53x10^{-3}	1.22x10^{-3}	[1.13x10^{-3}, 5.92x10^{-3}]	29926	2.88	.004 **
**Random Effects**						
	Variance			*SD*		
Participant (Intercept)	7.85x10^{-5}			8.86x10^{-3}		
**Model Fit**						
	Marginal			*Conditional*		
R^2	2.3x10^{-3}			0.02		
REML criterion	- 69453.80					

Note. Model: WTC ~ puzzles given + condition*puzzles correct + (1|id). *P*-values for fixed effects calculated using Satterthwaite's method, and confidence intervals and *p*-values computed used a Wald *t*-distribution approximation. *** $p < .001$, ** $p < .01$, * $p < .05$.

Table S12: HbO_2 coherence and maternal stress.

**Fixed Effects**						
	Estimate	*SE*	*95% CI*	*df*	*t*	*p*

Intercept	0.30	1.07×10^{-2}	[0.28, 0.32]	43337	28.18	< .001 ***
FullCollaboration	2.37×10^{-2}	5.68×10^{-3}	[0.01, 0.03]	43337	4.18	< .001 ***
CollaborationScreen	2.60×10^{-2}	5.69×10^{-3}	[0.01, 0.04]	43337	4.57	< .001 ***
Maternal Stress	2.81×10^{-4}	2.72×10^{-4}	[-2.52×10^{-4}, 8.15×10^{-4}]	43337	1.03	.306
FullCollaboration x Maternal Stress	-5.24×10^{-4}	1.47×10^{-4}	[-8.11×10^{-4}, -2.37×10^{-4}]	43337	-3.58	< .001 ***
CollaborationScreen x Maternal Stress	-5.55×10^{-4}	1.47×10^{-4}	[-8.43×10^{-4}, -2.67×10^{-4}]	43337	-3.78	< .001 ***
**Random Effects**						
	Variance			*SD*		
Participant (Intercept)	9.65×10^{-5}			9.82×10^{-3}		
**Model Fit**						
	Marginal			*Conditional*		
R^2	1.20×10^{-3}			0.02		
REML criterion	- 103350.90					

Note. Model: WTC ~ condition * maternal stress + (1|id). *P*-values for fixed effects calculated using Satterthwaite's method, and confidence intervals and *p*-values computed used a Wald *t*-distribution approximation. *** $p < .001$, ** $p < .01$, * $p < .05$.

Table S13: HbR coherence and maternal stress.

**Fixed Effects**						
	Estimate	*SE*	*95% CI*	*df*	*t*	*p*
Intercept	0.31	1.02×10^{-2}	[0.29, 0.33]	43337	30.54	< .001 ***
FullCollaboration	-1.44×10^{-2}	5.80×10^{-3}	[-0.03, -2.99×10^{-3}]	43337	-2.48	.013 *
CollaborationScreen	1.37×10^{-2}	5.82×10^{-3}	[2.31×10^{-3}, 0.03]	43337	2.36	.018 *
Maternal Stress	7.79×10^{-5}	2.59×10^{-4}	[-4.30×10^{-4}, 5.86×10^{-4}]	43337	0.30	.765
FullCollaboration x Maternal Stress	4.09×10^{-4}	1.50×10^{-4}	[1.15×10^{-4}, 7.03×10^{-4}]	43337	2.73	.006 **
CollaborationScreen x Maternal Stress	-3.52×10^{-4}	1.50×10^{-4}	[-6.47×10^{-4}, -5.80×10^{-5}]	43337	-2.35	.019 *
**Random Effects**						
	Variance			*SD*		
Participant (Intercept)	8.49×10^{-5}			9.22×10^{-3}		
**Model Fit**						
	Marginal			*Conditional*		
R^2	7.05×10^{-4}			0.02		
REML criterion	- 101435.10					

Note. Model: WTC ~ condition * maternal stress + (1|id). *P*-values for fixed effects calculated using Satterthwaite's method, and confidence intervals and *p*-values computed used a Wald *t*-distribution approximation. *** $p < .001$, ** $p < .01$, * $p < .05$.
